# The small heat shock protein αB-Crystallin protects versus withaferin A-induced apoptosis and confers a more metastatic phenotype in cisplatin-resistant ovarian cancer cells

**DOI:** 10.1371/journal.pone.0281009

**Published:** 2023-01-26

**Authors:** Melissa M. Carmichael, Israa Alchaar, Kathleen A. Davis, Merideth Kamradt Krevosky

**Affiliations:** Department of Biological Sciences, Bridgewater State University, Bridgewater, Massachusetts, United States of America; Washington State University, UNITED STATES

## Abstract

Since a majority of ovarian tumors recur in a drug-resistant form leaving patients few treatment options, the goal of this study was to explore phenotypic and molecular characteristics of a cisplatin-resistant ovarian cancer cell line (OVCAR8R) as compared to its cisplatin-sensitive syngeneic counterpart (OVCAR8) and to explore the effectiveness of a novel chemotherapeutic, Withaferin A (WA). In addition to unique morphological characteristics, the small heat shock proteins (Hsps) αB-Crystallin (HspB5) and Hsp27 are constitutively expressed along with increased expression of vimentin in OVCAR8R cells, while OVCAR8 cells do not endogenously express these Hsps, supporting that Hsp overexpression may confer resistance to chemotherapy and promote more aggressive tumor types. WA increases apoptosis in a dose-dependent manner in OVCAR8 cells, while OVCAR8R cells remain more viable at comparable doses of WA coincident with the upregulation of αB-Crystallin. To determine the significance of αB-Crystallin in conferring a more aggressive phenotype, αB-Crystallin was silenced by CRISPR-Cas9 in OVCAR8R cells. The morphology of the OVCAR8R clones in which αB-Crystallin was silenced reverted to the morphology of the original cisplatin-sensitive OVCAR8 cells. Further, cisplatin-resistant OVCAR8R cells constitutively express higher levels of vimentin and migrate more readily than cisplatin-sensitive OVCAR8 and OVCAR8R cells in which αB-Crystallin was silenced. Transient overexpression of wildtype αB-Crystallin, but not a chaperone-defective-mutant, alters the morphology of these cells to closely resemble the cisplatin-resistant OVCAR8R cells and protects versus WA-induced apoptosis. Together, this research supports the potential effectiveness of WA as a therapy for ovarian cancer cells that have not yet acquired resistance to platinum-based therapies, and importantly, underscores that αB-Crystallin contributes to a more aggressive cellular phenotype and as such, may be a promising molecular target for a better clinical outcome.

## Introduction

Ovarian cancer is the fifth leading cause of cancer-related deaths among women in the United States and is the deadliest malignancy amongst solid gynecological tumors [1]. The poor prognosis for ovarian cancer is partly attributed to that a majority of patients present with advanced disease, for which the standard treatment is surgery and platinum-based or taxane-based chemotherapy [2, 3]. While initial recurrence is frequently cisplatin-sensitive, patients often develop resistance to cisplatin treatment [4]. Despite initial response to treatment, most ovarian cancer patients develop recurrent disease with a drug-resistant phenotype within three years [1, 4, 5]. The initial response to platinum-based chemotherapy in ovarian cancer is considered refractory, resistant, or responsive to platinum-based chemotherapy. While these considerations are clinically important, the biological mechanisms that underlie cellular responses to chemotherapy are unclear [4]. Cancer cell resistance to platinum-based chemotherapies may be intrinsic, present before treatment, acquired, or induced by therapy. Resistance mechanisms, whether intrinsic or acquired, pose a major clinical problem in the treatment of ovarian cancer and are the main contributing factor in cancer-associated mortality. As such, a better understanding of acquired chemoresistance is clinically important [2]. Numerous mechanisms of platinum-resistance have been identified including enhanced DNA repair, disruption of apoptosis, reduced platinum accumulation, and the intracellular inactivation of cisplatin [2, 3]. Resistant cells acquire one or more mechanisms contributing to the multifactorial nature of cisplatin resistance [2, 3, 6, 7]. A prominent tool to gain insight to resistance mechanisms is the selection of drug-resistant lines derived from increasing drug concentrations [8]. Chowanadisai and Messerli, et al. derived a cisplatin-resistant ovarian cancer cell line (OVCAR8R) through exposure of OVCAR8 cells to sub-lethal concentrations of cisplatin [8]. In this model, resistance coincides with increased expression of proteins that underlie several hallmarks of cancer, including anti-apoptotic proteins, and proteins that are linked to a more mesenchymal phenotype [8]. Further, the resistance-derived gene signature of the OVCAR8R was correlated with that of patients with shorter survival.

Herein, we further investigate the mechanisms that underlie resistance to platinum-based therapies and hypothesize that the cisplatin-resistant phenotype confers additional therapeutic resistance to the OVCAR8R cells. While platinum-based drugs (specifically, cisplatin) are the standard of care and effective in the treatment of ovarian cancer [6], plant constituents, known as phytochemicals, have gained the status of clinically useful modern medicines and have been used for treating various diseases including malignant tumors [7]. Numerous phytochemicals are under investigation that may be employed as a secondary treatment to target cisplatin-resistant cells in various malignancies. The use of phytochemicals in the treatment of ovarian cancer holds promise as they may synergize with or target resistant cells with potentially fewer adverse side effects than platinum-based chemotherapies. *Withania somnifera* is a well-known Ayurvedic medicinal plant originating from Asia and Africa [9, 10]. Withaferin A, (WA), the principal active-phytochemical of *W*. *somnifera*, has broad therapeutic applications, including anti-inflammatory activity, modulation of the immune system, and an emerging effect on cancer inhibition as withanolides have proven to be cytotoxic to cancer cells [9, 10]. WA has promise as a cancer therapeutic given that it prevents tumor growth, angiogenesis, and metastasis, and induces apoptosis in diverse cancer cell types including lung, breast, prostate, colorectal and ovarian cancer [11]. Kakar *et al*. demonstrated that WA promotes apoptosis in ovarian cancer cell lines, and the combination of WA and cisplatin reduces the dose of cisplatin or doxorubicin required to achieve therapeutic effects. Therefore, this phytochemical holds potential to serve as a treatment for cisplatin-resistant ovarian cancers [11]. Despite this, the molecular mechanism underlying WA-induced apoptosis in chemoresistant ovarian cancer cells was not addressed. Apoptosis, or programmed cell death, is a tightly regulated molecular mechanism by which cells are eliminated by either intracellular- (intrinsic pathway) and extracellular-mediated (extrinsic pathway) signals [12, 13]. Disruption of apoptosis can occur through a variety of mechanisms causing tumor initiation, progression, and resistance to treatment. Much research has shown heat shock proteins (Hsps) bind to and inhibit several molecules that play critical roles in coordinating the process of apoptosis [12–15]. Hsps are stress-inducible molecules known for their chaperone activities that confer cell survival and proteostasis, including promotion of protein folding, cellular protein trafficking, and preventing the aggregation of client proteins [13–15]. Small Hsps are chaperones with a molecular weight of 12–43 kDa, which homo- or hetero-oligomerize with family members to carry out their chaperone function and often prevent apoptosis [12]. Hsp27 and αB-Crystallin (also known as HSPB5), have been shown to prevent apoptosis in several types of cancer cells [14, 15]; Hsp27 interacts with cytochrome *c* in the cytoplasm inhibiting the formation of the apoptosome, therefore, averting apoptosis while αB-Crystallin interacts with procaspase-3 inhibiting the activation of caspase-3, in turn, inhibiting apoptosis [13–16]. αB-Crystallin has been characterized as an oncoprotein that predicts poor clinical outcome in several solid tumors, including carcinomas of the breast, prostate, ovary, colon, liver, and its expression correlates with metastatic disease [14, 16–21]. αB-Crystallin is induced by matrix detachment and subsequently prevents anoikis, or anchorage-dependent cell death, and promotes lung metastasis in triple negative breast cancer cells [17]. αB-Crystallin increases invasion and metastasis of colorectal cancer cells and silencing of this protein reduces migration, increases apoptosis, and decreases tumorigenicity [18]. Several studies support that high expression of αB-Crystallin correlates with a poor prognosis for ovarian cancer patients and prevents chemotherapy-induced cell death [19–21]. To address the role of Hsps in conferring resistance to chemotherapy-induced apoptosis, we employed two matched ovarian cancer cell lines, which we show differentially express small Hsps. Our work supports that Hsp27 and αB-Crystallin are molecular markers of chemoresistance in ovarian cancer, and that cisplatin-resistance confers secondary chemotherapeutic resistance to WA. Concurrently, we show that WA leads to upregulation of αB-Crystallin in cisplatin-resistant cells and silencing of this protein restores apoptosis and inhibits cellular migration. Taken together, our studies support that αB-Crystallin promotes chemoresistance and migration and prevents apoptosis in ovarian cancer cells, underscoring its potential as a therapeutic target for a better clinical outcome.

## Materials and methods

### Cell lines and maintenance

The ovarian cancer cell lines, OVCAR8 (cisplatin-sensitive) and OVCAR8R (cisplatin-resistant) were provided by Dr. Shanta Messerli and were characterized as described previously [8]. Briefly, the human ovarian adenocarcinoma cancer cell line OVCAR8 cell line (originally purchased through the National Cancer Institute Developmental Therapeutics Program’s tumor repository program) was made to be cisplatin-resistant through stepwise exposure to cisplatin up to 5 μM to create the cisplatin-resistant cell line OVCAR8R. Cells were generously provided to us and OVCAR8R cells were maintained in 5μM Cisplatin (Sigma) dissolved in 0.9% NaCl to continue selection pressure of this resistant cell line. Cells were maintained in Dulbecco’s Modified Eagle Medium (DMEM, Sigma) supplemented with 10% fetal bovine serum and 1% penicillin streptomycin. Cells were maintained and incubated at 37°C in a 5% CO_2_ environment.

### Withaferin A treatment

Withaferin A (EMD Millipore) was diluted in DMSO according to the manufacturer’s instructions. Cells were plated 24 h prior to treatment at a concentration of 250,000 cells/mL in six well plates. Cells were treated with concentrations of 0, 1.0, 2.5, 5.0 and 7.5μM Withaferin A, then allowed to incubate for 24 h prior to collection for each experimental procedure as described below.

### Annexin and viability staining and flow cytometry

For Annexin V-FITC staining, cells were treated as described, after which adherent and non-adherent cells were collected and washed in 1 mL of 1x Binding Buffer (Miltenyi Biotechnology) at 300 *x g* for 10 minutes. The cell pellet was resuspended in 100 μL of 1x Binding Buffer and 10μL of Annexin V-FITC was added prior to a 15-minute incubation in reduced lighting. Cells were washed in 1 mL of 1x Binding Buffer at 300 *x g* for 10 minutes. The cell pellet was resuspended in 500 μL of 1x Binding Buffer. 5 μL of Propidium Iodide (PI) solution was added immediately prior to analysis by flow cytometry via MACSQuant (Miltenyi). Data was analyzed using the MACSQuantify Software to determine percentage of cells that were Annexin positive.

For viability staining, adherent and nonadherent cells were pooled after treatment with WA as described. Cells were pelleted at 300 *x g* for 10 minutes and were resuspended in 100 μL of 1x PEB Buffer (PBS, 0.5% bovine serum albumin, and 2 mM EDTA); 1μL of Viobility ™ 488/520 fixable dye (Miltenyi Biotechnology) was added and incubated for 15-minutes in reduced lighting. Cells were washed in 1 mL of PBS and collected at 300 *x g* for 10 minutes. The cell pellet was resuspended in 500 μL of 1x PEB with 5 μL of Propidium Iodide (PI) solution immediately prior to analysis by flow cytometry via MACSQuant (Miltenyi). Viable cells are reported as those that exclude Viobility dye and PI. Data was analyzed using the MACSQuantify Software to determine percentage of cells that were Annexin positive.

### Cell harvest and Western blot analysis

Cells were treated as described, after which adherent and non-adherent cells were collected for protein analysis. Cells were scraped using a cell scraper, then cells were centrifuged, washed in PBS with PMSF/Protease Inhibitors and re-suspended in an equal volume of PBS and 2x Laemmli buffer. DNA was sheared using a 26-gauge needle and 10 μL lysate were loaded into each well of a 15% polyacrylamide gel. SDS-PAGE was performed on whole-cell lysates and proteins were transferred to a PVDF membrane. The membrane was blocked in blocking buffer (5% milk in Tris-Buffered Saline Tween-20, TBST) for 1 hour. Membranes were incubated for 1 hour each in primary antibodies diluted in 1% milk in TBST. Primary antibodies were used at the following dilutions 1:1000 dilution of Hsp27, or alpha-B Crystallin (Enzo), 1:2500 dilution of anti-Tubulin (Sigma, T5168), or 1:1000 dilutions of Vimentin or PARP-1 (Cell Signaling Technologies). The membrane was washed four times in TBST for 5 minutes and incubated at room temperature for 1 hour on a rocker in 10 mL of secondary antibody solutions (1:10,000 secondary HRP-conjugated anti-mouse or anti-rabbit antibodies (Cell Signaling Technologies) in 1% milk in TBST. The membrane was washed four times with TBST for 5 minutes, then once in dH_2_O for 5 minutes, followed by incubation in 10 mL ECL Chemiluminescence for 2 minutes and exposed to film. For expression of αB-Crystallin and densitometry, Image J was used to find values for each band; expression levels for αB-Crystallin were normalized to tubulin and the ratio of WA-induced αB-Crystallin expression was compared to that of untreated cells.

### Immunocytochemistry

Cells were plated on coverslips in six well plates and allowed to adhere overnight. For cytoskeletal staining, cells were fixed in 0.25% Glutaraldehyde/2% Paraformaldehyde for 15 minutes and coverslips were incubated with a FITC-conjugated antibody to detect vimentin (Alexa Fluor® 488 Conjugate-Vimentin, Cell Signaling Technologies) for 30 minutes. Coverslips were washed by dipping in PBS, then incubated for 15 minutes with DyLight™ 554 Phalloidin (Cell Signaling Technologies) to detect actin. Coverslips were washed by dipping in PBS. After a final wash in water, coverslips were placed on slides with mounting media containing DAPI (Life Technologies).

### Puromycin death curve

OVCAR8R cells were plated in twenty-four well plates at a density of 40,000 cells/well. Twenty-four hours after plating, cells were treated with increasing concentration of puromycin (Santa Cruz) with doses ranging from 0–10.0 μg/ml. Detached cells and media were removed every three days and the same concentration puromycin were added to each well to establish the lowest dose at which no cells remained viable to allow selection of cells that express a puromycin-resistance gene. OVCAR8R cells were selected in 0.5μg/mL puromycin and individual clones were isolated.

### Transfection of CRISPR plasmids and selection of knockout clones

OVCAR8R cells were transfected with 1 μg of CRISPR αB-Crystallin-GFP vector, 1 μg of CRISPR alpha-B Crystallin HDR-RFP vector or 1 μg of control vector (pEGFP-N1, CLONTECH) and allowed to recover for 48 h. Cells were then transferred to 150mm plates and clones stably expressing these constructs were selected by growth in 0.5μg/mL puromycin (Life Technologies) for 3 weeks. Individual colonies were selected and rescued using sterile cloning cylinders. Clones were trypsinized from each cloning cylinder sealed with vacuum grease and puromycin-resistant clones were propagated and examined for expression of αB-Crystallin by immunoblotting with a monoclonal antibody for αB-Crystallin (Enzo) as described above.

### Transient transfection experiments for analysis of apoptosis or morphological analysis

Cells were plated on coverslips and then transiently transfected with GFP or FLAG-tagged wild-type αB-Crystallin or a FLAG-tagged triple pseudophosphorylation mutant (S19E,S45E,S59E, 3XSE) as performed in prior work [16]. After overnight incubation, cells were treated with DMSO (control) or 2.5 μM WA for 24 h. Following treatment, GFP-transfected cells were fixed in 0.25% Glutaraldehyde/2% Paraformaldehyde for 15 minutes and coverslips were placed on slides with mounting media containing DAPI (Life Technologies). FLAG-vector transfected cells were fixed in 100% cold methanol and analyzed by immunofluorescence using an anti-FLAG mAb (SIGMA) and anti-mouse FITC antibody (SIGMA) and coverslips were placed on slides with mounting media containing DAPI (Life Technologies). The morphology of transfected control or WA-treated cells was analyzed for morphological characteristics and for apoptotic nuclei. The percentage of nontransfected or GFP- or FLAG-tagged wild-type αB-Crystallin or FLAG-tagged 3XSE transfectants was scored and data represent the mean of apoptotic cells with the standard error of three or four independent experiments. Significance was determined by analysis by ANOVA and a follow up paired two-tailed Student’s *T* Test with data reported as significant as indicated by asterisks (*, p≥ 0.05, **p≥ 0.02).

### Transwell migration assay

OVCAR8 (cisplatin-sensitive) and OVCAR8R (cisplatin-resistant) cells and the CRISPR-Clones 1A or 7A in which αB-Crystallin was silenced (CrαB1A or CrαB7A), were plated at a density 75,000 cells per well in the top chamber of a six well plate with a transwell insert (8.0 mm, Corning) that had been equilibrated for a minimum of 2h in 10% FBS-containing DMEM. DMEM with 20% FBS was added to the bottom chamber to promote cellular migration and cells were incubated for 24 h, after which nonmigrating cells were removed from the top of the membrane with a cotton swab. Cells that had migrated through the transwell membrane to the lower chamber were washed twice in PBS, fixed in 100% methanol, and stained with 0.25% Crystal Violet in 20% methanol. Stained cells that had migrated to the lower chamber were viewed using a 10X objective, eight images per well were captured and Image J was used to score percent migration as relative units per field for each cell line. The data is represented as mean of eight fields per experiment for three independent experiments with the standard error of the mean expressed.

## Results

### Morphological and protein analysis of OVCAR8 and OVCAR8R cell lines

**Fig 1:**
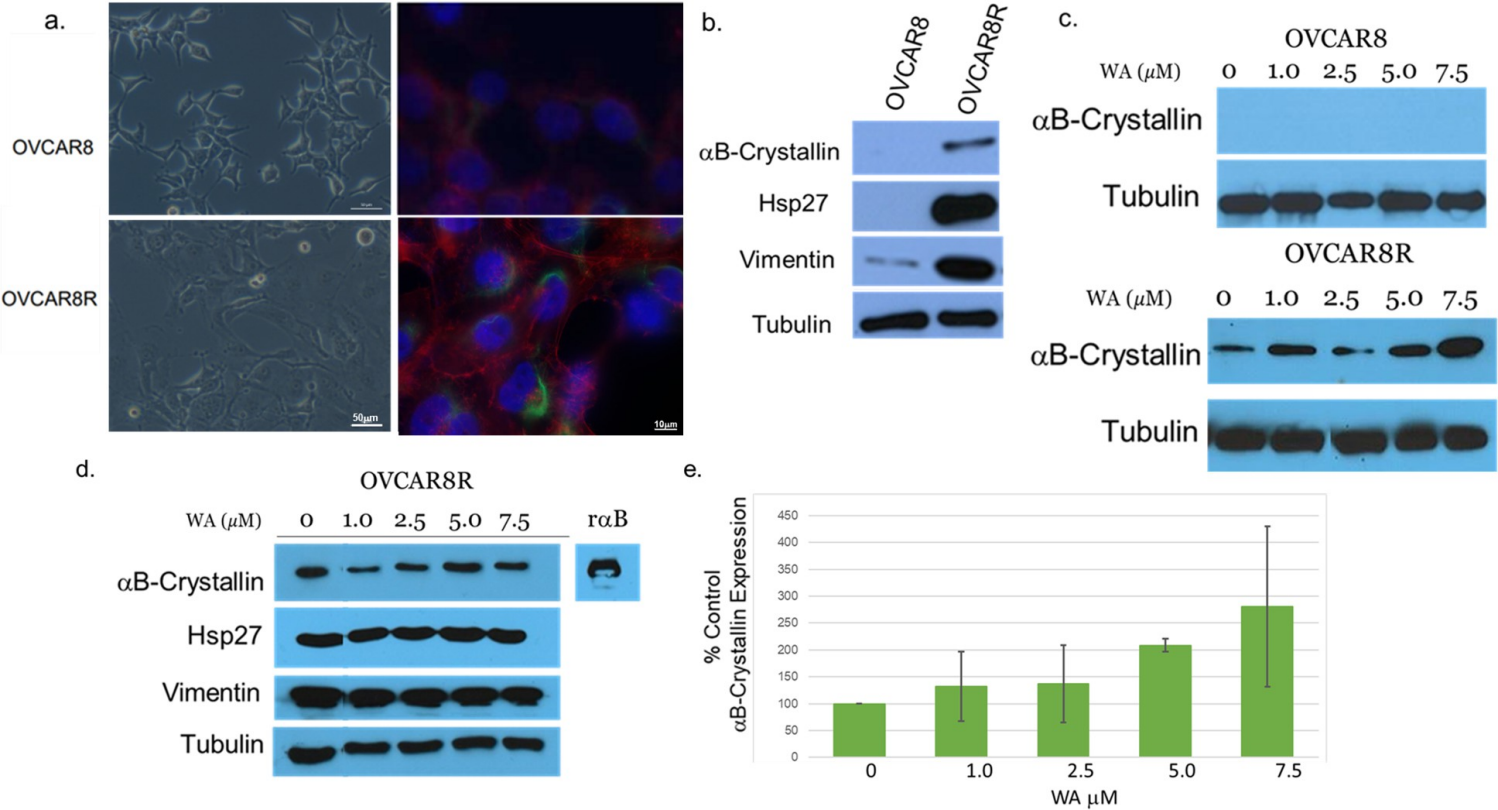
Cisplatin-resistant OVCAR8R cells demonstrate a unique morphology and more metastatic protein signature. (a) Morphological comparison of OVCAR8 (upper panels) and OVCAR8R cells (lower panels) as imaged by phase contrast (left panels) or via fluorescence microscopy targeting the cytoskeletal proteins actin (red) and vimentin (green) via immunocytochemistry and DNA via DAPI (blue). OVCAR8R cells appear flatter and darker and show more extensive cytoskeletal staining as compared to OVCAR8 cells; (b, c, d) Protein from total cell lysates from OVCAR8 or OVCAR8R cells was analyzed via Western Blot analysis for expression of Hsps, and Vimentin. Tubulin expression shows equal protein is loaded in each well. (c, d) Cells were incubated for 24h with vehicle (DMSO), 1.0μM, 2.5 μM 5.0μM or 7.5μM WA, after which protein from total cell lysates was analyzed via Western Blot analysis. (e) Mean expression of αB-Crystallin was determined by densitometric analysis using Image J. The expression as normalized to tubulin ± SE is graphed for three independent experiments; a linear regression reveals a significant positive correlation between increased dose of WA and increased expression of αB-Crystallin (p≥ 0.005, r^2^ = 0.485).

In order to characterize the morphological and molecular traits involved in the resistance of cells to cisplatin, OVCAR8 and OVCAR8R cells were analyzed for cellular morphology via immunocytochemistry for actin and vimentin or expression of Hsps and Vimentin. (Fig 1A and 1B). Consistent with the findings of our colleagues who created these syngeneic matched cell lines, in monolayer cultures, OVCAR8R cells show greater adhesion and a darker and flatter appearance using phase contrast microscopy, whereas the Cisplatin-Sensitive OVCAR8 cells appear smaller and are more spindle-shaped (Fig 1A). Immunocytochemistry for actin and vimentin supports this morphological characterization of each cell line in that the cisplatin-resistant OVCAR8R cells display a more extensive cytoskeleton with marked extensions of actin (red) and vimentin (green) filaments as compared to the more condensed, spindle-shaped cisplatin-sensitive OVCAR8 cells.

Molecular analysis by Western blot shows that expression of Hsp27 and αB-Crystallin were not detected in the OVCAR8 cell line, however, both Hsp27 and αB-Crystallin are constitutively expressed in OVCAR8R (Fig 1B). Hsp overexpression correlates with higher expression of vimentin, a well-characterized marker that supports a more metastatic phenotype of the chemoresistant cells (OVCAR8R) (Fig 1). To determine the effect of WA on protein expression, cells were incubated with 1.0μM, 2.5μM, 5.0μM or 7.5μM WA for 24hrs, after which expression of Hsp27, Vimentin and αB-Crystallin were analyzed as compared to tubulin as a loading control (Fig 1C, 1D). While expression of Hsp27 and Vimentin are not altered in OVCAR8R cells in response to WA, we demonstrate a dose-dependent upregulation of αB-Crystallin in OVCAR8R, but not OVCAR8 cells when treated with WA (recombinant αB-Crystallin is used as a control). Densitometric analysis reveals a significant dose-dependent upregulationin expression of αB-Crystallin after treatment with WA as compared to untreated cells (Fig 1E). These data support that this small heat shock protein is induced by a secondary chemotherapeutic agent which may thereby promote further chemoresistance.

### Morphological and flow cytometric analysis of WA-treated OVCAR8R cells

**Fig 2:**
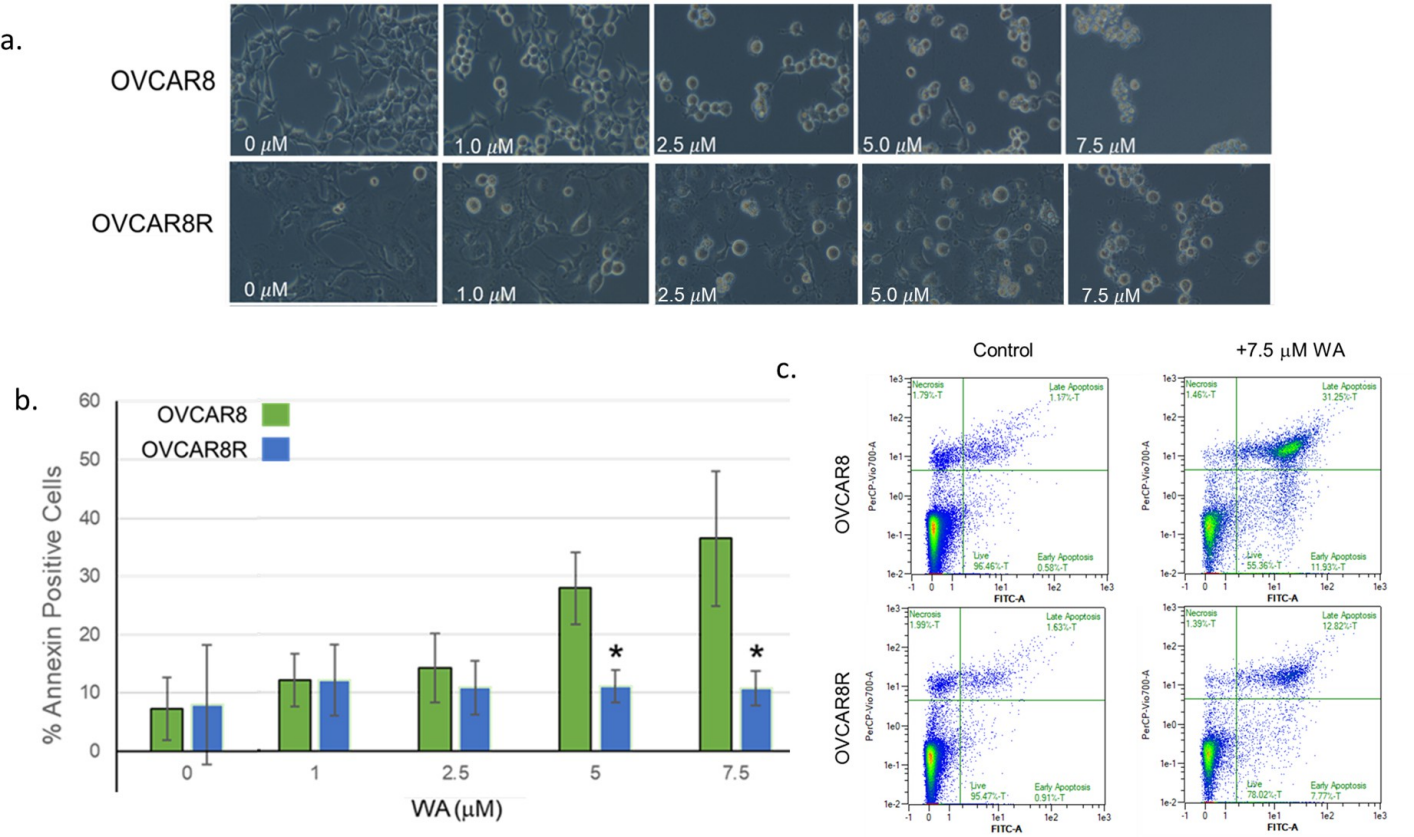
OVCAR8R are more resistant than OVCAR8 cells to WA-induced apoptosis via morphology and flow cytometry. (a) Morphological analysis of cells incubated with vehicle (DMSO), 1.0μM WA, 2.5μM WA, 5.0μM WA, or 7.5μM WA. OVCAR8 cells detach from the plate with increasing concentrations of WA (a, top panels), while OVCAR8R cells exhibit less cell detachment at comparable concentrations of WA (a, bottom panels). (b) The percent Annexin positive cells is graphed ± SE for three independent experiments. Concentrations of 5.0 μM and 7.5μM WA induce a significant increase in Annexin positive cells in OVCAR8 cells, but not in the OVCAR8R cells. ANOVA and a two-tailed Student’s *T* Test were run to determine significance; statistical comparisons (* p≥ 0.05) indicate a significant difference in percent Annexin positive cells between OVCAR8 and OVCAR8R cells after WA treatment at concentrations of 5.0μM and 7.5μM WA. (c) A representative analysis shows flow cytometry results from OVCAR8 cells (upper panels) or OVCAR8R cells (lower panels) treated with vehicle (DMSO, left) or 7.5 μM WA (right) after 24 h. Cells were stained with Annexin V-FITC and PI. The percent apoptotic cells were graphed as the total percentage of cells that are positive for Annexin V-FITC (Lower and Upper Right Quadrants).

To determine the effect of WA on cell survival, cells were incubated with, 1.0μM, 2.5μM, 5.0μM, or 7.5μM WA for 24hrs. Cellular morphology shows WA-induced cytotoxicity in both OVCAR8 and OVCAR8R cells at the highest dose of 7.5 μM WA(Fig 2A, 2B). While WA is cytotoxic to both cell lines at high concentrations, the OVCAR8R cells retain more viability and are still adhered to the plate at 2.5μM and 5.0 μM WA while few to no OVCAR8 cells appear viable and retain attachment to the plate at these doses (Fig 2A). Annexin V-FITC staining was performed to determine the percent of cells that had externalized phosphatidylserine, a hallmark of apoptosis. Flow cytometry reveals that the percent of Annexin positive cells increases in a dose-dependent manner in OVCAR8 cells following WA treatment, while the percent of Annexin positive OVCAR8R cells is significantly decreased in comparison at the dose of 5.0 μM and 7.5 μM WA (* p≥ 0.05) (Fig 2B, 2C). A representative image of flow cytometry results is shown to compare the OVCAR8 to OVCAR8R cells at 5.0 μM WA (Fig 2C). These results demonstrate that the cells that have acquired resistance to cisplatin have also acquired more general chemoresistance given their decreased sensitivity to WA-induced apoptosis.

### Morphological and molecular characterization of OVCAR8, OVCAR8R and αB-Crystallin knockout clones

**Fig 3:**
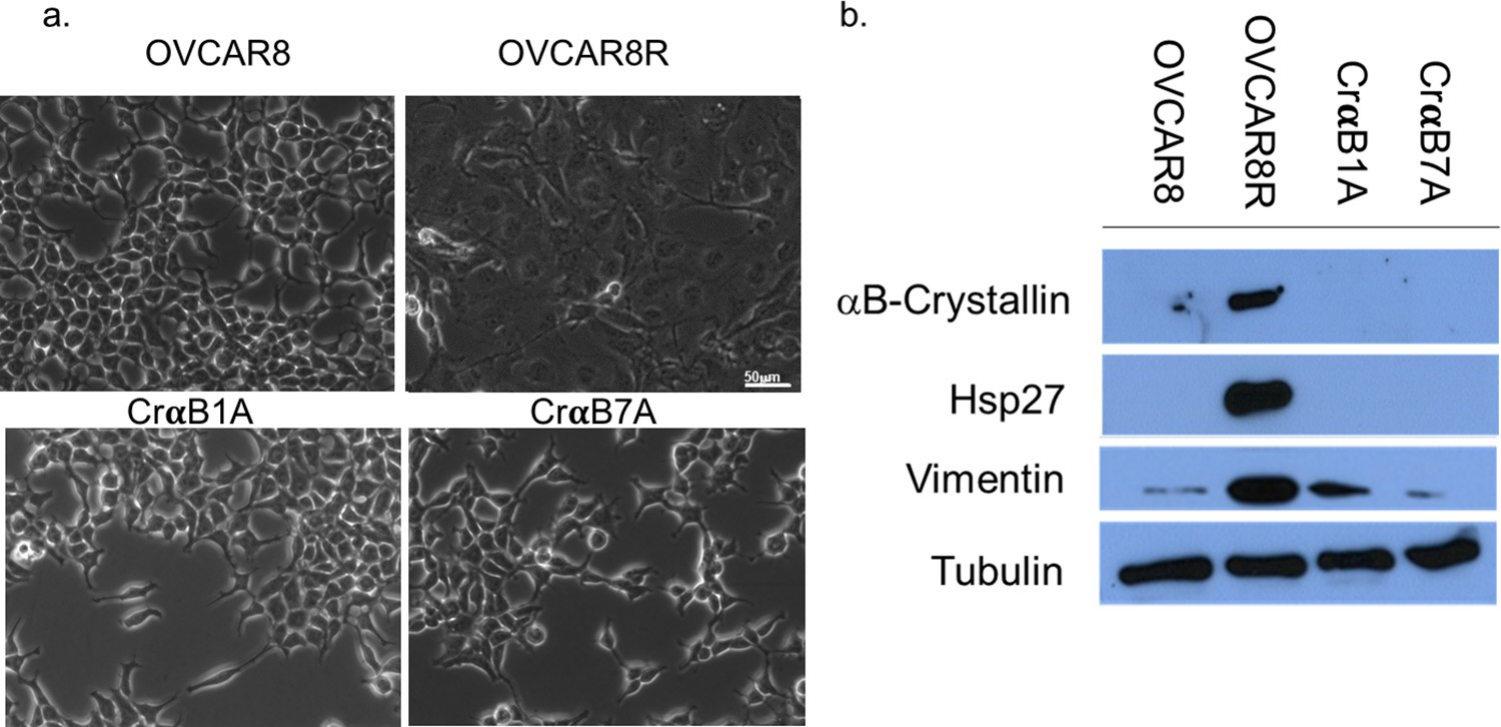
OVCAR8R cells show similar morphological and molecular characteristics to OVCAR8 cells when αB-Crystallin is silenced. (a) αB-crystallin was silenced by CRISPR-Cas9 in OVCAR8R cells. The morphology of the OVCAR8R-αB-crystallin-knockout clones (CrαB1A and CrαB7A) revert to the morphology of the cisplatin-sensitive OVCAR8 cell line from which the OVCAR8R cells were originally derived as shown by phase contrast microscopy. (b) Protein from total cell lysates from OVCAR8, OVCAR8R, CrαB1A and CrαB7A cells was analyzed via Western Blot analysis for expression of αB-Crystallin, Hsp27 and vimentin. Molecular characterization reveals downregulation of vimentin expression in the αB-Crystallin-null OVCAR8R clones CrαB1A and CrαB7A. Tubulin expression shows that equal protein is loaded in each well.

To determine whether αB-Crystallin plays a role in conferring chemoresistance, αB-Crystallin was silenced through the use of a CRISPR-Cas9 expression construct in OVCAR8R cells. Western blot analysis confirmed fifteen successful αB-Crystallin knockout clones, with two clones (CrαB1A and CrαB7A) selected for the further study. Notably, all of the clones in which αB-Crystallin was silenced show morphological and molecular characteristics similar to OVCAR8 cells, losing the phenotype of the OVCAR8R cells (Fig 3A, 3B). Morphological analysis (CrαB1A and CrαB7A shown here) reveals the clones return to the cisplatin-sensitive cell morphology of OVCAR8 cells and lose the cisplatin-resistant morphology of OVCAR8R they had prior to selection. This finding supports that αB-Crystallin is likely to be an important player in the morphological conversion of OVCAR8 cells as they gained resistance to cisplatin. In order to analyze expression of other mediators of apoptosis and metastasis, protein was isolated from OVCAR8, OVCAR8R and the αB-Crystallin knockout clones CrαB1A and CrαB7A. Expression of Hsp27 was downregulated in the αB-Crystallin null cells along with a downregulation of vimentin, a hallmark protein involved in metastasis. In order to ensure that targeting of αB-Crystallin did not impact Hsp27 expression, we confirmed that Hsp27 expression remained inducible following CRISPR-Cas9 targeting of αB-Crystallin; heat shock experiments reveal that when CrαB1A and CrαB7A are exposed to heat stress at 42°C for one hour, Hsp 27 was upregulated 24 h after heat shock while no expression of αB-Crystallin was detected (S1 Fig). Together, this work supports that loss of αB-Crystallin is coincident with a less aggressive cellular and molecular phenotype wherein the cells display a morphology similar to the cisplatin-sensitive cell type decrease expression of metastatic markers.

### Ectopic expression of wildtype αB-Crystallin alters the morphology of ovarian cancer cells

**Fig 4:**
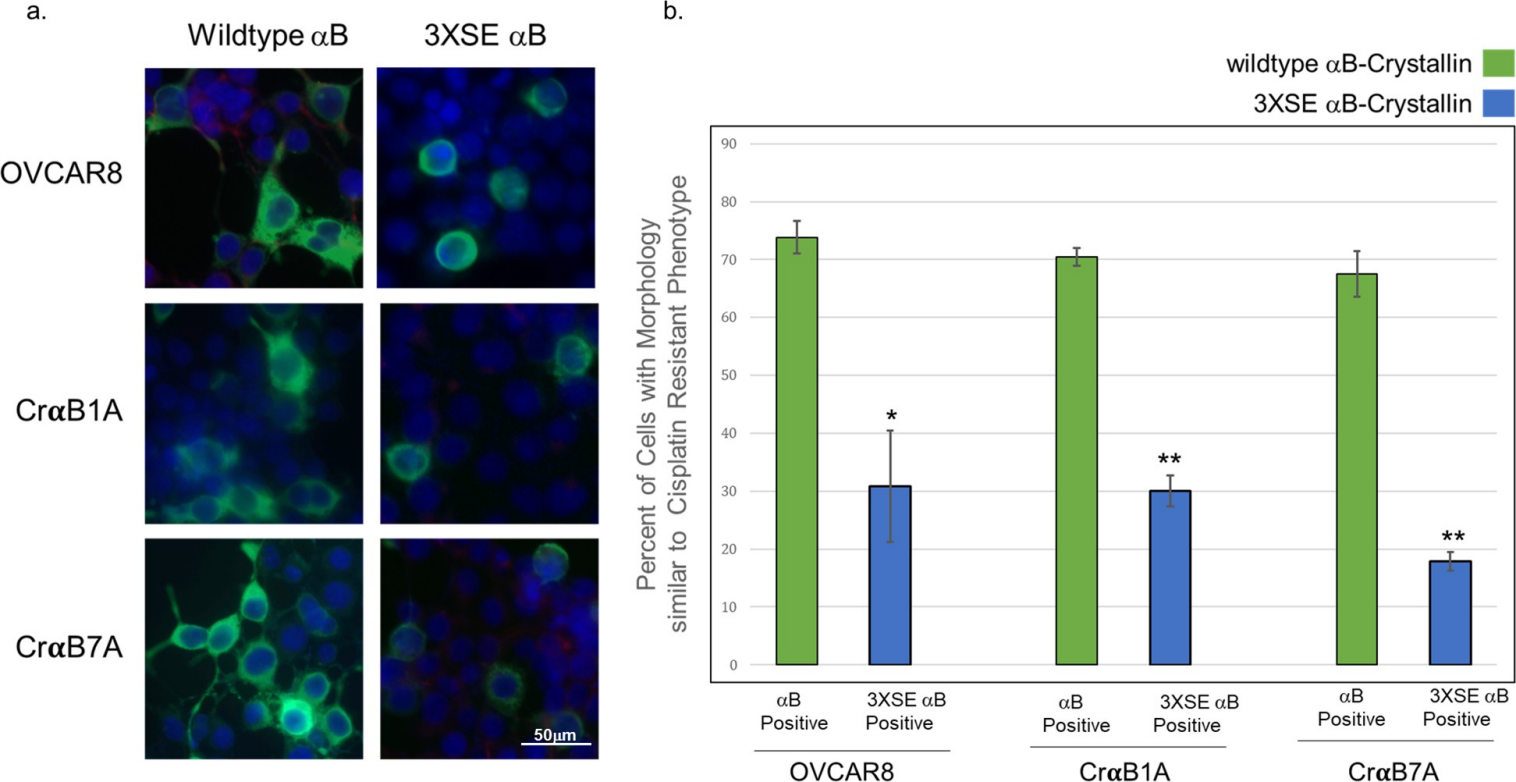
Transient overexpression of wildtype αB-Crystallin, but not a chaperone-defective mutant, confers a flattened cellular morphology. (a) Cells were transiently transfected with empty vector or FLAG-tagged wild-type αB-Crystallin (left panels) or the pseudophosphorylation mutant (3XSE, right panels). The percentage of transfected cells with a flattened, cobblestone-like morphology consistent with that of the OVCAR8R cells were scored. (b) OVCAR8 Cells or the αB-Crystallin-null OVCAR8R clones CrαB1A and CrαB7A were scored for the percentage of flattened cells; wildtype αB-Crystallin expression (green bars) significantly promotes a cobblestone-shaped morphology as compared to chaperone-defective mutant, 3XSE-transfected cells (blue bars). The data represent the mean ± SE of three independent experiments for each cell line. ANOVA and a two-tailed Student’s *T* Test were run to determine significance; statistical comparisons (* p≥ 0.05, **p≥ 0.02) indicate a significant difference in percent cells that morphologically resemble OVCAR8R for wildtype αB-Crystallin (green bars) as compared to 3XSE-transfected (blue bars) cells for each cell line.

To address the role of αB-Crystallin on the morphological change noted in the knockout clones, transient transfection experiments were conducted using the OVCAR8 or OVCAR8R cells in which αB-Crystallin was silenced (CrαB1A and CrαB7A). OVCAR8, CrαB1A or CrαB7A cells were transiently transfected with FLAG-tagged wildtype or chaperone-defective αB-Crystallin a after which FLAG-expressing cells were identified via immunocytochemistry and transfected cells were scored for their morphology. In all three cell types, αB-Crystallin expressing-cells show a morphology similar to OVCAR8R cells, rather than the phenotype of the OVCAR8 cells (Fig 4A, 4B). Indeed, of those cells that transiently overexpress αB-Crystallin, a flattened, cobblestone-like morphology (green bars) similar to those of the OVCAR8R cells was found in 73.9%, 70.5% or 67.5% for OVCAR8, CrαB1A and CrαB7A, respectively. Morphological analysis of cells expressing the 3XSE (blue bars) mutant show significantly less cells have the morphology of the cisplatin-resistant cells in comparison to wildtype αB-Crystallin (green bars), suggesting that chaperone activity underlies this characteristic. Our results support that expression of wildtype αB-Crystallin confers a morphology similar to the more chemoresistant phenotype while the pseudophosphorylation mutant does not. Significance was determined by analysis by ANOVA and a follow up paired two-tailed Student’s *T* Test between wildtype and 3XSE αB-Crystallin for each cell line, data reported as significant as indicated by asterisks (* p≥ 0.05, **p≥0.02).

### αB-Crystallin promotes migration in ovarian cancer cells

**Fig 5:**
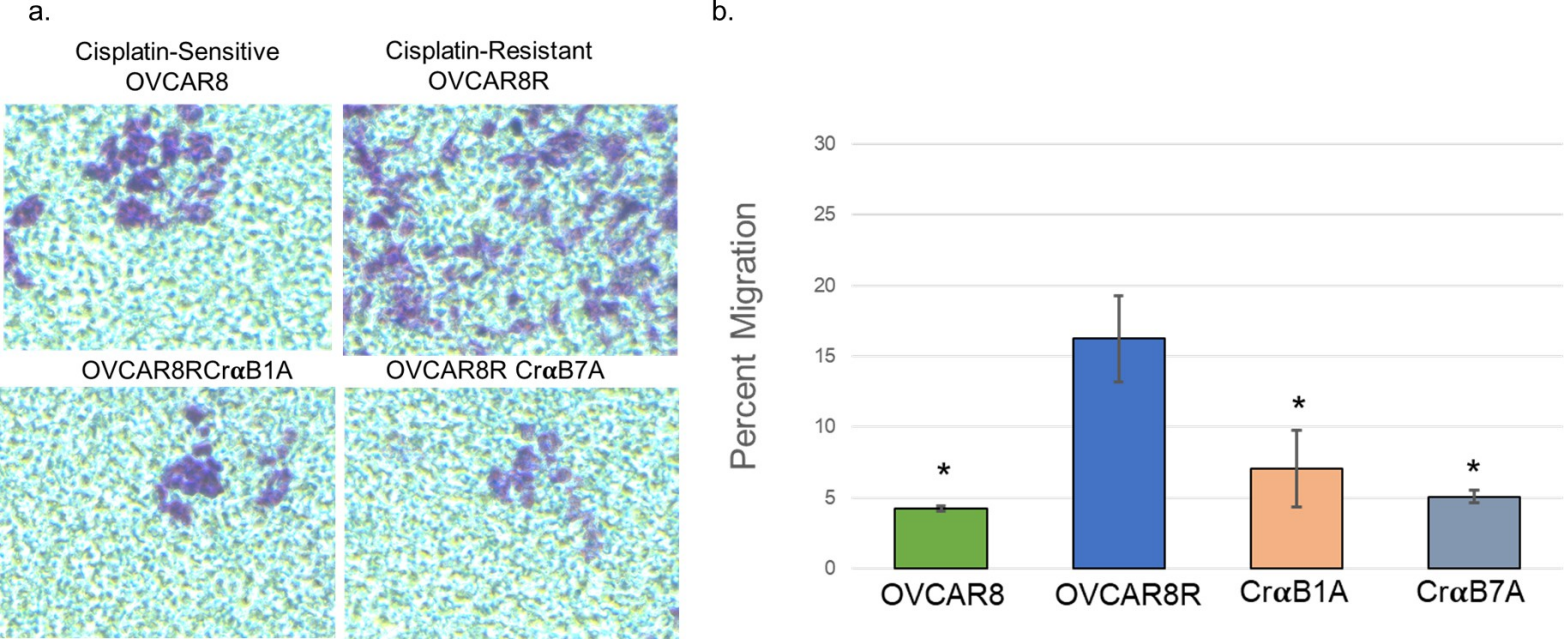
Expression of αB-Crystallin promotes the migration of ovarian cancer cells. Percent migration of cells through a transwell membrane was measured; cells were plated, allowed to migrate toward 20% FBS in the bottom chamber, then fixed and stained with Crystal violet after 24 h. (a) A representative image of migrated cells (OVCAR8, OVCAR8R, CrαB1A or CrαB7A) as imaged on the underside of the transwell membrane, 24 h after plating. (b) A significant increase in migration was noted for the OVCAR8R cells through the transwell membrane as compared to the OVCAR8 cells and the αB-Crystallin null cells (CrαB1A and CrαB7A). Eight fields in each of three independent experiments were quantified for each cell type. The data represent the mean ± SE of three independent experiments for each cell line. Significance was determined by ANOVA and a follow up paired two-tailed Student’s *T* Test between the four cell lines; data is reported as significantly decreased migration in each cell line as compared to the OVCAR8R cells as indicated by asterisks (* p≥ 0.05).

The changes noted in morphology and protein expression in the αB-Crystallin-null cells reveal that they may possess a more mesenchymal phenotype, and as such, may be better capable of migration. As shown by Chowanadisai and Messerli, the serial selection of cisplatin resistant cells led to an increase in molecular markers that support a more mesenchymal expression profile [8]. Microarray analysis of over 3100 genes revealed that several genes of interest in this study were differentially expressed in the OVCAR8R cells as compared to the OVCAR8 cells. Furthermore, the gene expression signature that differentiate OVCAR8 and OVCAR8R cells include markers that are associated with patient survival, supporting that molecular features of OVCAR8 versus OVCAR8R cells reflect patient tumor behavior. Of interest, molecular markers of a more invasive phenotype were upregulated in the chemoresistant cells (OVCAR8R) as compared the chemosensitive (OVCAR8) cells, and include matrix metalloprotease (7-fold), fibronectin (6-fold) and vimentin (4-fold). The small heat shock proteins αB-Crystallin and Hsp27 were upregulated (by 5.5-fold and 2.4-fold, respectively) [8], a finding that is directly supported by of our results (Fig 1B). Therefore, to further characterize the role of αB-Crystallin in promoting a more mesenchymal, metastatic phenotype, we investigated whether cellular migration was coincident with increased expression of αB-Crystallin, Hsp27 and vimentin (Figs 3B, 5). In order to determine the role of αB-Crystallin in migration of cells, OVCAR8, OVCAR8R and the CRISPR-αB-Crystallin null clones (CrαB1A and CrαB7A) were plated on a transwell insert and allowed to migrate using 20% FBS as a chemoattractant in the bottom chamber to promote cellular migration. A representative image shows that after 24 h, OVCAR8R cells migrate more readily in comparison the OVCAR8, CrαB1A and CrαB7A) cells, supporting that αB-Crystallin expression promotes migration (Fig 5A). Indeed, a significant increase in migration of OVCAR8R cells (16.7%) was observed as compared to the cisplatin-sensitive OVCAR8 cells (4.3%) and the OVCAR8R cells in which αB-Crystallin was silenced by CRISPR-Cas9, CrαB1A (7.0%) and CrαB7A (5.1%). Eight fields from each of three independent experiments were quantified for each cell type. Significance was determined by analysis by ANOVA and a follow up paired two-tailed Student’s *T* Test between each cell line, data reported as significant as indicated by asterisks (* p≥ 0.05). Together, these data support that αB-Crystallin not only confers chemoresistance, but also promotes migration, which may support a more metastatic and aggressive phenotype in ovarian cancer cells.

### WA induces downregulation of vimentin in αB-Crystallin null chemoresistant ovarian cancer cells

**Fig 6:**
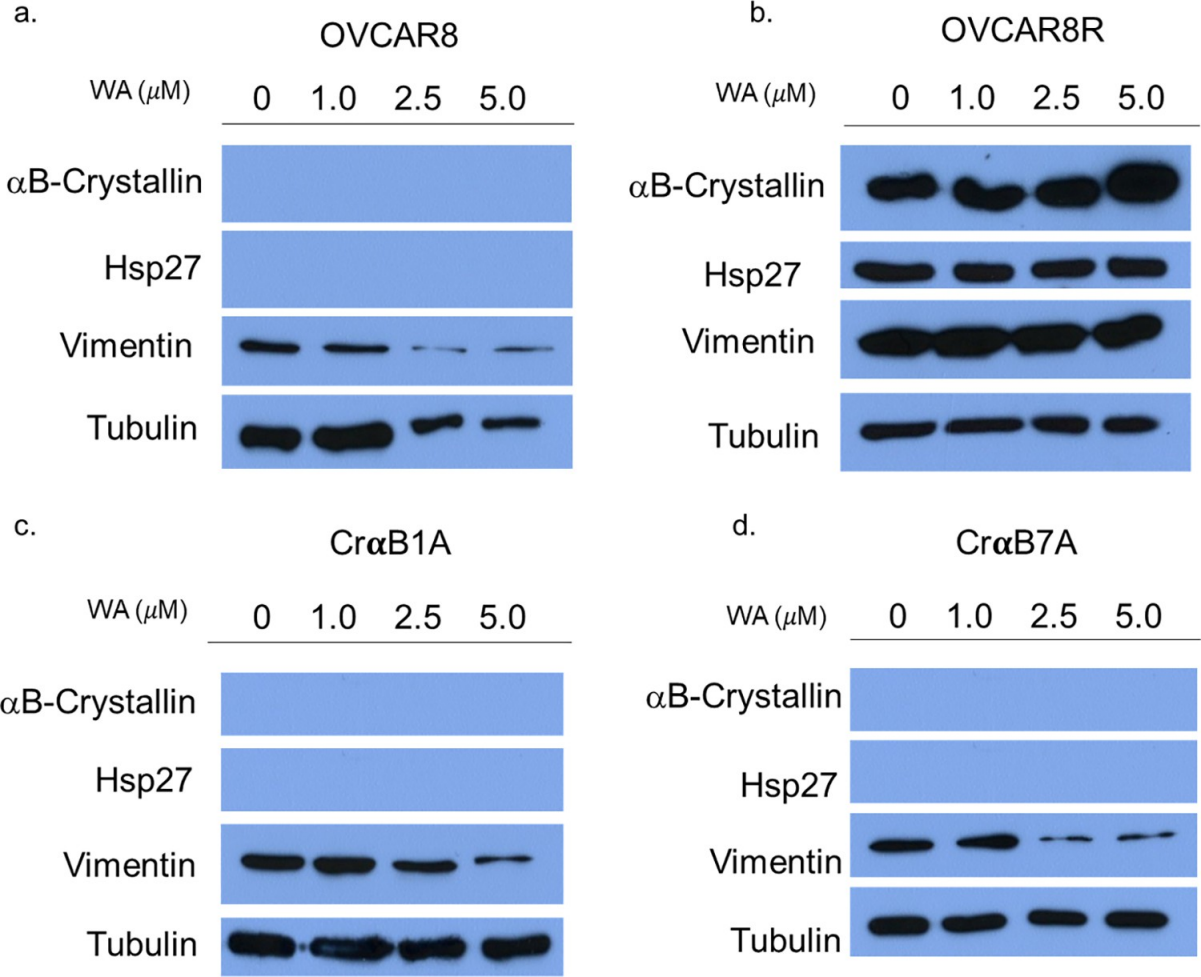
WA-induced downregulation of vimentin expression occurs in ovarian cancer cells which lack αB-Crystallin. Protein from total cell lysates from cells incubated with vehicle (DMSO), 1.0μM WA, 2.5μM WA, or 5.0μM WA was analyzed via Western Blot analysis. Expression of αB-Crystallin, Hsp27 and vimentin are shown in OVCAR8 (a), OVCAR8R (b), CrαB1A (c) and CrαB7A (d) cells. Tubulin expression shows equal protein is loaded in each well.

Several models using diverse cancer types have demonstrated that Withaferin A treatment inhibits experimental epithelial to mesenchymal transition (EMT), and inhibition of this event may be attributed to its impact on vimentin assembly [22–25]. Vimentin is an intermediate filament essential for cell motility and migration [24]. Overexpression of vimentin correlates with more metastatic disease, EMT induction, and poor prognosis; several studies support that WA treatment suppresses expression of vimentin and initiates its disassembly [24–26]. Loss of vimentin expression nearly eliminates cell migration demonstrating the therapeutic potential of WA as an anti-metastatic agent [24, 25]. OVCAR8 or OVCAR8R cells in which αB-Crystallin was silenced (CrαB1A and CrαB7A) show a dose-dependent downregulation of vimentin following treatment with WA (Fig 6A, 6C, 6D). In contrast, the chemoresistant OVCAR8R cells do not downregulate vimentin following treatment with WA, concurrent with an upregulation of αB-Crystallin, as noted previously (Figs 1C–1E and 6B). This supports that expression of αB-Crystallin and vimentin in OVCAR8R cells likely confer a more metastatic and mesenchymal phenotype as supported by our migration studies (Fig 5) and as such, are more difficult to target with anti-metastatic agents.

### Ectopic expression of wildtype αB-Crystallin protects ovarian cancer cells from WA-induced apoptosis

**Fig 7:**
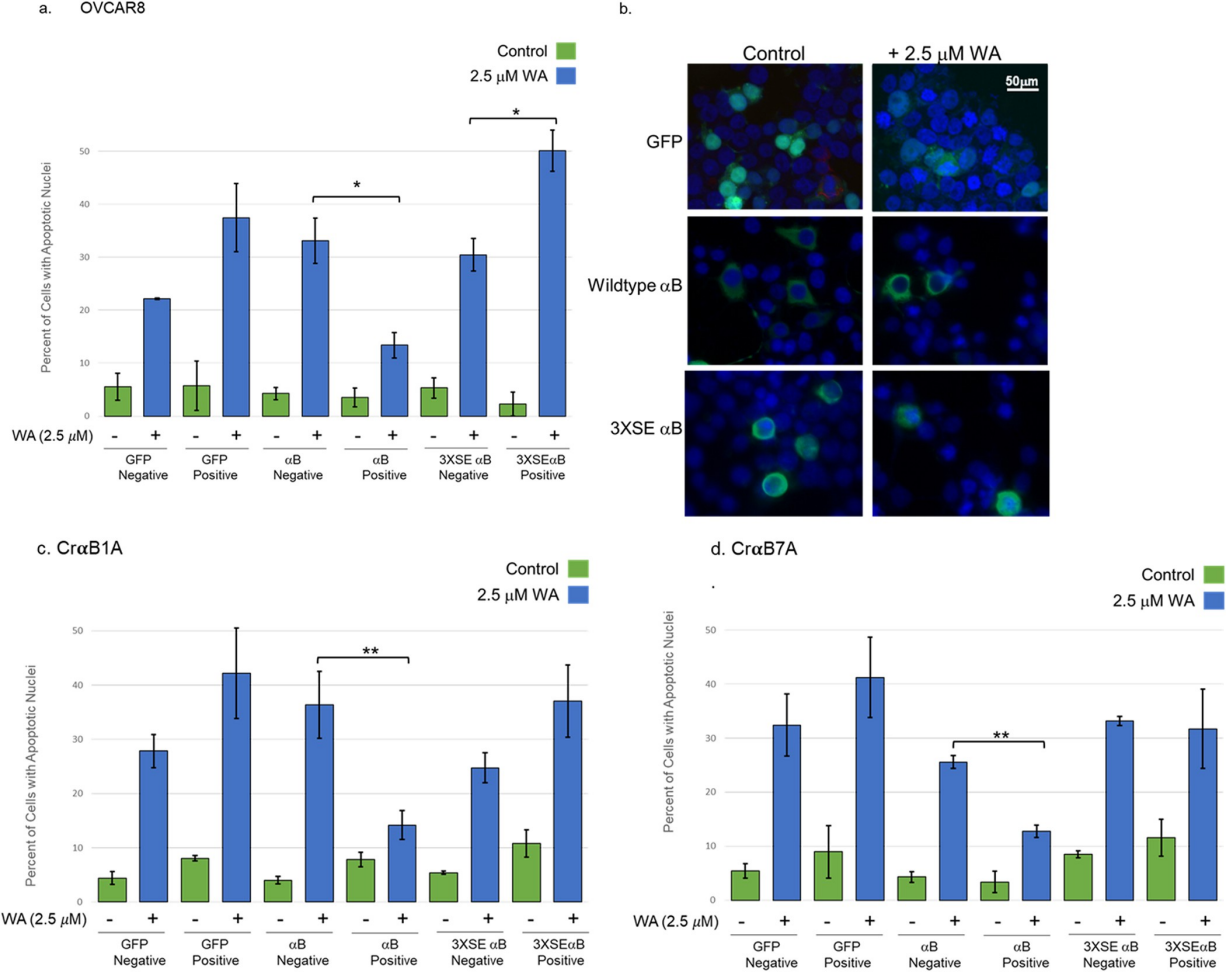
Ectopic expression of αB-Crystallin protects ovarian cancer cells from WA-induced apoptosis. (a) OVCAR8 Cells were transiently transfected with GFP vector or FLAG-tagged wild-type αB-Crystallin or the αB-Crystallin pseudophosphorylation mutant (3XSE). After overnight incubation, cells were treated with 2.5μM WA for 24 h, coverslips were fixed and stained. Nontransfected and transfected cells were scored for their apoptotic morphology (condensed or fragmented nuclei) in control (green bars) versus WA-Treated (blue bars) cells. (b) A representative photomicrograph of OVCAR8 cells following fixation of cells is shown; micrographs show expression of GFP (top), or wildtype αB-Crystallin (middle) or 3XSE (bottom) αB-Crystallin. The left panels show control cells while the right panels show cells treated with 2.5μM WA. Condensed and/or fragmented nuclear morphology of cells are noted in the right panel, and cells with nuclei corresponding to GFP- or αB-Crystallin-positive cells indicated by green fluorescence were scored as transfected cells. Percent of apoptotic cells are shown in control (green bars) versus WA-Treated (blue bars) CrαB1A cells (c) or CrαB7A cells (d). The data represent the mean ± SE of three or four independent experiments for each cell line. Significance was determined by ANOVA and a follow up paired two-tailed Student’s *T* Test between each construct as analyzed between control versus WA-treated cells in each of the three cell lines; data is reported as significant as indicated by asterisks (*p≥ 0.05, * *p ≥0.02).

To directly address the role of αB-Crystallin on the inhibition of WA-induced apoptosis, transient transfection experiments were conducted using ovarian cancer cells lacking endogenous αB-crystallin, OVCAR8 or OVCAR8R cells (CrαB1A and CrαB7A) cells in which αB-Crystallin was silenced. OVCAR8R cells were not used in this study given their high constitutive expression of αB-Crystallin, therefore overexpression through transient transfection experiments were not conducted with this cell line. OVCAR8, CrαB1A or CrαB7A cells were plated on coverslips and transiently transfected with GFP or FLAG-tagged wildtype αB-Crystallin or the chaperone-defective, mutant αB-Crystallin (3XSE). At 24h following transfection, cells were treated with DMSO (control) or 2.5 μM WA for 24 h; the dose of 2.5 μM WA was chosen for the treatment of transfected cells as treatment with higher doses (5.0 or 7.5 μM WA) yielded too few viable cells to score via immunocytochemistry. Cells were subsequently examined for expression of GFP or FLAG (wildtype or mutant αB-Crystallin) expression and scored for apoptosis based on nuclear morphology. The percentage of nontransfected and transfected (GFP, FLAG-tagged wildtype or 3XSE αB-Crystallin) cells undergoing apoptosis is reported for control (green bars) and WA-treated (blue bars) cells. Nontransfected cells were scored for their apoptotic morphology via nuclear DAPI staining, while the nuclei of transfected cells were identified by green fluorescence (GFP- or via immunofluorescence using an anti-FLAG mAb and FITC-conjugated secondary antibody) (Fig 7). Transient overexpression of wildtype αB-Crystallin protects all three αB-Crystallin-null ovarian cancer cell types in which it was overexpressed from WA-induced apoptosis. Indeed, in OVCAR8 cells (Fig 7A, 7B) as well as in CrαB1A (Fig 7C) and CrαB7A (Fig 7D), cells expressing wildtype αB-Crystallin show significant inhibition in WA-induced apoptosis. In contrast, transient expression of the triple pseudophosphorylation αB-Crystallin mutant (3XSE) did not protect cells from cytotoxicity as induced by WA (CrαB1A and CrαB7A) or by contrast, led to an increase in apoptosis (OVCAR8 cells), (Fig 7A, 7C, 7D). The data represent the mean of apoptotic cells and SE of three or four independent experiments. Significance was determined by analysis by ANOVA and a follow up paired two-tailed Student’s *T* Test between each treatment group, with data reported as significant as indicated by asterisks (* p≥ 0.05, **p≥0.02). Together, these data support that expression of αB-Crystallin confers resistance to WA-induced apoptosis while the chaperone-defective αB-Crystallin did not protect cells from apoptosis and may sensitize cells to apoptosis.

### Silencing of αB-Crystallin expression in OVCAR8R cells restores sensitivity to apoptosis and decreases viability following WA treatment

**Fig 8:**
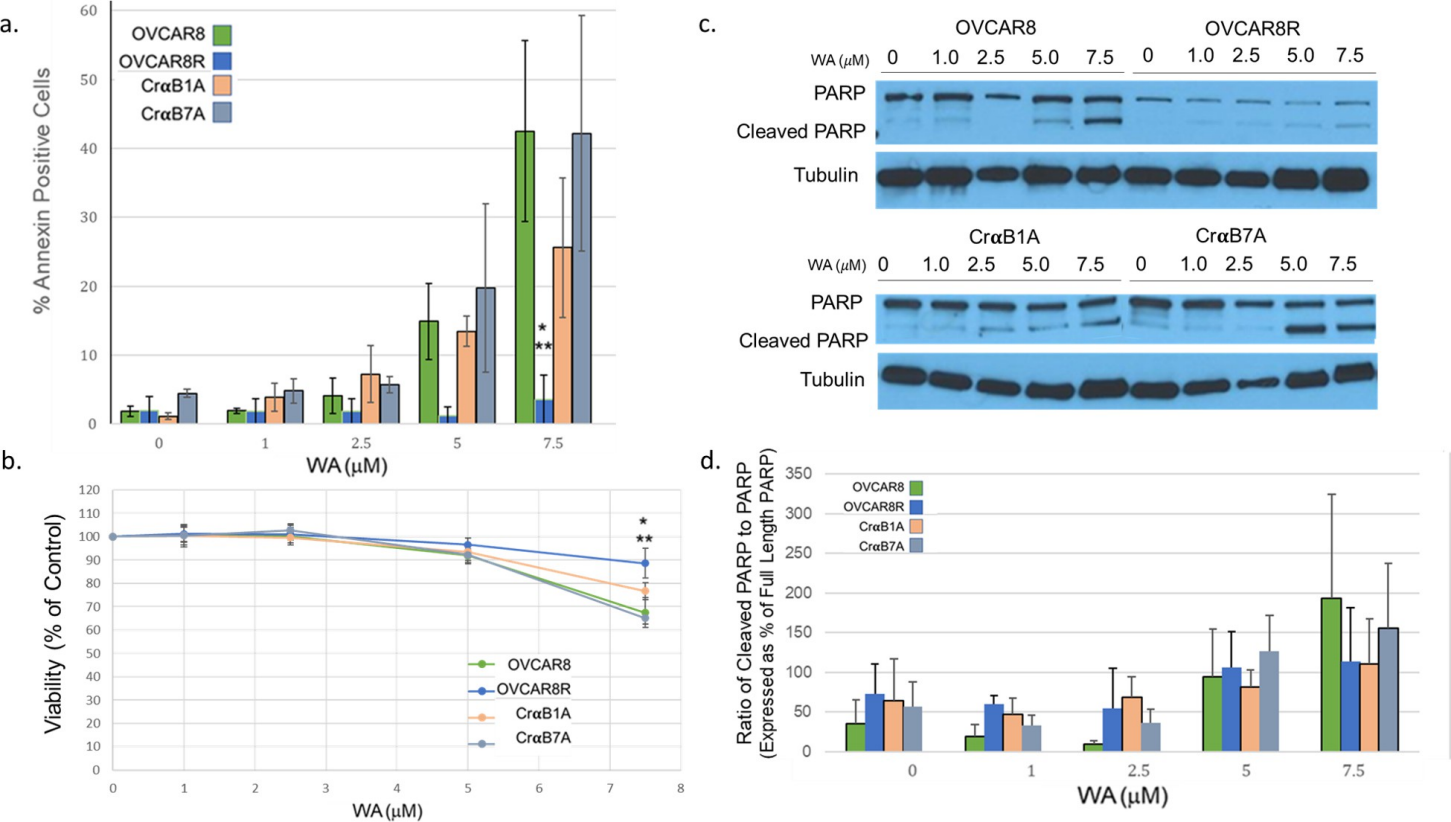
OVCAR8R cells are more resistant to WA-induced apoptosis and are more viable than OVCAR8, CrαB1A and CrαB7A cells when challenged with WA. (a) Flow cytometric analysis was performed after staining of cells for Annexin V-FITC and PI after cells were incubated for 24h with vehicle (DMSO), 1.0μM WA, 2.5μM WA, 5.0μM WA, or 7.5μM WA. The percent Annexin positive cells is graphed ± SE for three independent experiments. At a concentration of 7.5μM WA, a significant increase in Annexin positive cells is observed in OVCAR8, CrαB1A and CrαB7 cells in comparison to OVCAR8R cells. A two-factor ANOVA and a Sidak follow up test were run to determine significance; statistical comparisons indicate a significant difference in percent Annexin V-FITC positive cells between OVCAR8R cells and either OVCAR8 and CrαB7A (** p≥ 0.001) or CrαB1A cells (* p≥ 0.02) after WA treatment at concentration of 7.5μM WA. (b) Viability was determined by flow cytometry after treatment with WA and expressed as the percent of cells excluding PI and Viobility (Miltenyi) and normalized to the control (DMSO). There was a significant increase in viability between OVCAR8R cells and either OVCAR8 and CrαB7A (** p≥ 0.001) or CrαB1A cells (* p≥ 0.02) after WA treatment at concentration of 7.5μM WA. (c) Western blot analysis of PARP cleavage after treatment with WA (a representative image from OVCAR8 and OVCAR8R cells (upper panels) or CrαB1A and CrαB7A cells (lower panels). (d) The ratio of cleaved PARP versus full length PARP was determined and expressed as a percent of full length PARP.

To directly address the role of αB-Crystallin on the inhibition of WA-induced apoptosis, experiments were conducted using the OVCAR8, OVCAR8R or OVCAR8R cells in which αB-Crystallin was silenced (CrαB1A and CrαB7A). Annexin V-FITC staining reveals that at the doses of 7.5 μM WA, OVCAR8R cells show fewer apoptotic cells as compared to OVCAR8 and CrαB7A cells (** p≥ 0.001) and CrαB1A cells (* p≥ 0.02). This effect was confirmed by analysis of PARP cleavage in which cleaved PARP was more abundant in OVCAR8, CrαB1A and CrαB7A cells at doses of 7.5 μM WA, and less so in OVCAR8R cells. Densitometric analysis to determine the ratio of cleaved to full length PARP was conducted for three independent experiments (Fig 8D); this data corresponds well with the Annexin V-FITC analysis showing more cleaved PARP in OVCAR8 and in CrαB7A cells as compared to the OVCAR8R cells. Analysis of cell viability was performed 24h after treatment with WA through staining with Viobility™ 488/520 Fixable Stain and PI; cells that exclude each stain are reported as viable and were normalized to the percent of the control DMSO-treated cells. Analysis reveals that OVCAR8R cells are significantly more viable than CrαB7A (* p≥ 0.02), OVCAR8 and CrαB1A cells (** p≥ 0.001) (Fig 8B). Significance for both Annexin V-FITC and viability staining was determined by a two factor ANOVA analysis which revealed a cell line and dose dependent difference in the percent of apoptosis or viability, respectively, with a follow up pairwise comparison using Sidak analysis between each treatment group.

## Discussion

αB-Crystallin is overexpressed in diverse types of solid tumors, including malignant glioblastomas, osteosarcoma, retinoblastoma and carcinomas of the breast, prostate, ovary, colon, and liver [14]. A study of tumors from platinum- or taxane-treated ovarian cancer patients shows increased expression of αB-Crystallin was significantly associated with poor patient outcomes and overall survival [21]. Another study demonstrates that co-expression of αB-Crystallin and p53 significantly increases recurrence, metastasis, and death in ovarian cancer patients [20]. However, other data show that downregulation of Hsps is associated with a poor clinical outcome in some ovarian cancer types, but this study did not directly address expression of αB-Crystallin [27, 28]. Our study supports that αB-Crystallin is an important oncoprotein that is induced in the conversion of OVCAR8 cells as they gain resistance to cisplatin. The chemoresistant OVCAR8R cells constitutively overexpress the small Hsps, αB-Crystallin and Hsp27, while OVCAR8 cells that respond to cisplatin treatment do not endogenously express these proteins. Few studies have addressed the role of WA on the expression of Hsps in cancer cells, with one study supporting that WA treatment led to upregulation of Hsp70 and HspA6 in prostate cancer cells, protecting against programmed cell death [29]. No studies have addressed the role of WA on expression of αB-Crystallin in cancer cells. Therefore, to further understand the impact of WA as a secondary chemotherapeutic on Hsp expression and induction of apoptosis in chemoresistant versus chemosensitive ovarian cancer cells, we investigated the expression of Hsp27 and αB-Crystallin following treatment with WA. While OVCAR8 cells undergo apoptosis in a dose-dependent manner, the chemoresistant OVCAR8R cells do not enter into apoptosis as readily concurrent with an upregulation of αB-Crystallin. This finding supports that those cells that gain chemoresistance may be those that upregulate these and other Hsps. Our findings support that introduction of exogenous wildtype αB-Crystallin in cells in which this protein was silenced through CRISPR-Cas9 confers resistance to WA-induced apoptosis. Importantly, only overexpression of wildtype αB-Crystallin but not a chaperone-defective mutant protects cells from WA-induced apoptosis, underscoring the importance of this small Hsp, and its interaction through homo- or heterodimerization in conferring a more chemoresistant phenotype. The mutant αB-Crystallin in which three serine residues are altered to glutamate to mimic phosphorylation prevents Hsp oligomerization; therefore, our work supports that chaperone function is necessary for conferring chemoresistance to WA. Together, these data provide a clinically relevant rationale to target this protein to restore the sensitivity of cells to chemotherapy-induced cell death.

Our study utilizes a novel cell line, the OVCAR8 cisplatin resistant cell line (OVCAR8R) derived by Chowanadisai and Messerli [8]. Genes differentially expressed between the parental and cisplatin resistant OVCAR8R cells reflect a more mesenchymal state and support that the OVCAR8R cell line may express proteins that underlie cisplatin resistance in ovarian cancer. Of over 3100 genes analyzed [8], the fold increase in expression of the small Hsps, αB-Crystallin, Hsp27 and Vimentin support the protein expression profile in our study. Of note, in these cell lines, similar to ovarian tumors, multiple mechanisms appear to contribute to resistance and the differentially expressed genes in the cisplatin resistant OVCAR8R cells correlate with poor prognostic markers in patients [8]. Indeed, OVCAR8R cells show changes in several genes associated with EMT and a more mesenchymal state, especially when grown as spheroids. While our work focused on the biology of the monolayer cells as compared to spheroids, our data support these observations that the OVCAR8R cells are more mesenchymal in nature based on their morphology, increased expression of vimentin, resistance to a secondary chemotherapeutic agent and increased migration capability.

Other proteins associated with a more mesenchymal and metastatic phenotype are MMPI and Fibronectin, two additional markers of metastatic potential. MMP1, a member of the Matrix metalloproteinase family of proteases that degrade the extracellular matrix during physiological processes, including embryonic development and tissue remodeling, have been subsequently found to be upregulated in nearly every tumor type [30–32]. MMPs proteolytically process substrates in the extracellular matrix, in turn, leading to tumor invasion and metastasis [30–32]. Fibronectin is a matrix glycoprotein that plays a role in cell growth, differentiation, and processes such as wound healing and blood coagulation [33]. Increased Fibronectin expression has been shown to mediate various metastatic-promoting mechanisms, including tumor growth and invasion [33, 34]. Together, the overexpression of genes that support a more aggressive and metastatic phenotype in the OVCAR8R cells warrants further study. Since the expression level αB-Crystallin was shown to be comparable to that of fibronectin, and more abundant than vimentin, well-known metastatic markers, its importance in resistance to cisplatin deserves further investigation [8]. While our study is limited to analysis of cells derived from only one parental cell line, former work supports that in these matched cells, the increased expression of mesenchymal markers and a gene expression signature associated with patient survival support that this model holds mechanisms relevant to patients.

Future studies will evaluate the impact of αB-crystallin on other cellular endpoints that address the hallmarks of cancer biology. Of note, cells in which αB-crystallin is silenced will be cultured to be resistant to anoikis through either suspension cultures or selection of anoikis-resistant cells after growth in low-adherence culture conditions. Indeed, αB-Crystallin was shown to be a novel regulator of anoikis-resistance as induced by matrix detachment, which led to suppression of ERK signaling underlying metastatic potential; as such, αB-crystallin is a promising molecular target for anti-metastatic therapies such as WA [17]. Biochemical processes that underlie the anticancer effects of WA include suppression of inflammatory pathways, selective inhibition of tumor cell proliferation and induction of apoptosis, suppression of tumor angiogenesis, blockade of EMT, tumor invasion and metastasis, alteration of tumor cell metabolism among other attributes [10, 11]. Altered cellular motility, a hallmark feature of metastasis, requires reorganization of the actin cytoskeleton which is for EMT and cancer progression [35]. Morphological analysis revealed that the OVCAR8R αB-Crystallin knockout clones, CrαB1A and CrαB7A, lose the cellular phenotype of the chemoresistant cells. The CrαB1A and CrαB7A shed the morphology of a larger, flatter cell with marked actin cytoskeleton similar to the cisplatin-resistant cells they had prior to the targeting and selection and revert to a more condensed morphology similar to the cisplatin-sensitive cell morphology. Transient overexpression of αB-Crystallin in OVCAR8, CrαB1A and CrαB7A alters cellular morphology so that cells exhibit a more cobblestone-like morphology that more closely matches that of the OVCAR8R cells. Since vimentin is key to the migratory phenotype and is implicated in chemoresistance and tumorigenicity, it is therefore a therapeutic clinical target to decrease metastatic potential of cancer cells [36]. Our study supports that cells that lack αB-crystallin also express lower levels of vimentin and do not migrate as readily as those cells that express this Hsp. Also, WA further downregulates vimentin with no change in vimentin expression in chemoresistant OVCAR8R that constitutively express αB-crystallin. Therefore, our study supports that use of the phytochemical WA may prevent metastasis in ovarian cancer cells. Future studies using the current cell lines include analyzing the angiogenic potential of each cell line in the presence of human vein endothelial cells (HUVECs) as well as the impact of silencing αB-crystallin on adhesion of each cell line to various extracellular matrix substrates (Matrigel, collagen, laminin). Former work shows that αB-crystallin promotes adhesion of breast cancer cells to diverse ECM proteins, supporting the importance of this protein in extravasation and support of micrometastases [37]. Studies are also underway to silence the closely related small heat shock protein Hsp27 to further explore its importance in resistance to apoptosis, cellular migration, adhesion and angiogenic potential in ovarian cancer cell lines. Future studies will address the impact of Hsps and WA on migration and invasion through use of additional cell lines in which αB-crystallin, Hsp27, or both small Hsps are silenced. Together, our study supports that investigation of Hsps are clinically relevant as markers of chemoresistance, and support that the cisplatin resistant OVCAR8R cell line model is relevant to ovarian cancer patients. Further investigation into the role of these chaperone proteins in the development of resistance in ovarian cancer is warranted. Our study supports the importance of Hsps in multidrug chemoresistance in ovarian cancer and supports the use of an *in vitro* model to gain new insights into cisplatin resistance. Taken together, this study underscores an important role of αB-Crystallin as well as the effectiveness of WA as a potential therapy for ovarian cancer cells that have not yet acquired resistance to platinum-based therapies.

## Supporting information

S1 Fig Heat shock experiments confirm successful silencing of αB-Crystallin in CrαB1A and CrαB7A. Heat shock of the OVCAR8, OVCAR8R, CrαB1A and CrαB7A at 42°C for 1h shows that Hsps are upregulated by 24 h after heat shock with no expression of αB-Crystallin detected in CrαB1A and CrαB7A cells.
(TIF)

S1 File Densitometry, apoptosis, morphology and viability data. (XLSX)

S1 Raw images (PDF)
