# Supplementary figures and images for: The small heat shock protein αB-Crystallin protects versus withaferin A-induced apoptosis and confers a more metastatic phenotype in cisplatin-resistant ovarian cancer cells
Source: PLoS One. 2023 Jan 26;18(1):e0281009. doi: 10.1371/journal.pone.0281009 (PMC9879449; doi:10.1371/journal.pone.0281009)

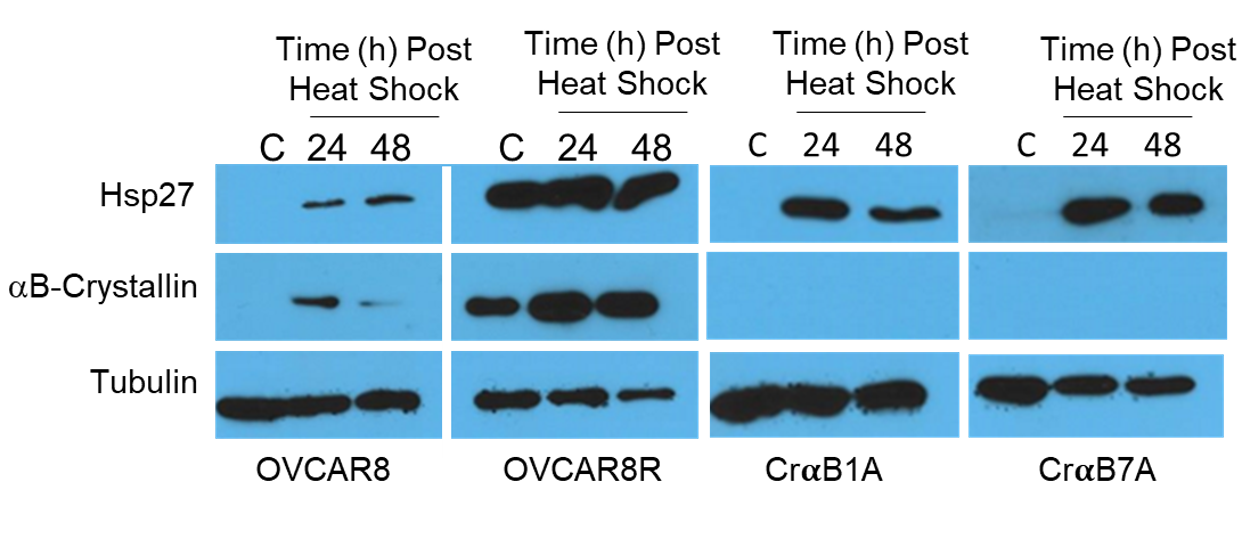

Supplement: S1 Fig — Heat shock of the OVCAR8, OVCAR8R, CrαB1A and CrαB7A at 42°C for 1h shows that Hsps are upregulated by 24 h after heat shock with no expression of αB-Crystallin detected in CrαB1A and CrαB7A cells. (TIF) [file pone.0281009.s001.tif]
