# Supplementary material for: The small heat shock protein αB-Crystallin protects versus withaferin A-induced apoptosis and confers a more metastatic phenotype in cisplatin-resistant ovarian cancer cells
Source: PLoS One. 2023 Jan 26;18(1):e0281009. doi: 10.1371/journal.pone.0281009 (PMC9879449; doi:10.1371/journal.pone.0281009)
Supplement: S1 Raw images — (PDF) [file pone.0281009.s003.pdf]

Films_Data _Fig 1b, 3b
Samples run in duplicate
Lanes 1-4 skip lane 5
Lanes 6-9 as shown in key

OVCAR8	OVCAR8R	CrαB1A	CrαB7A
1	2	3	4
6	7	8	9

LANES

b.

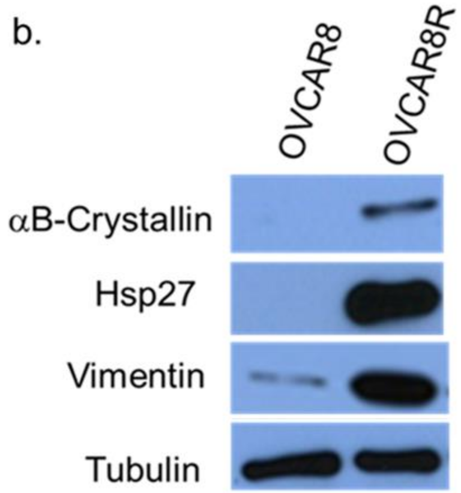

b.

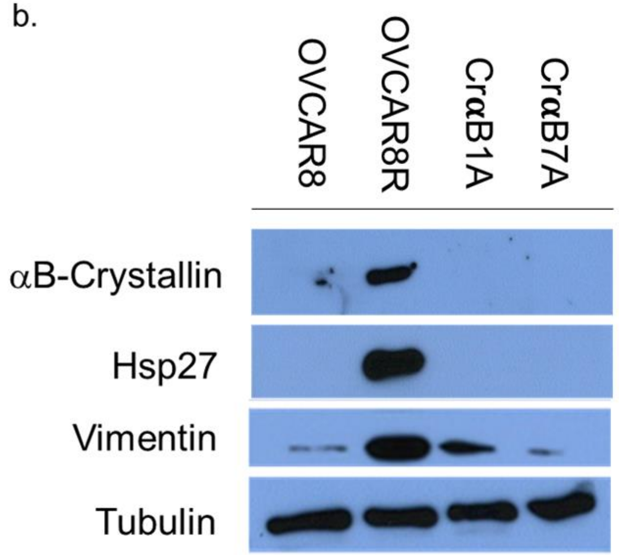

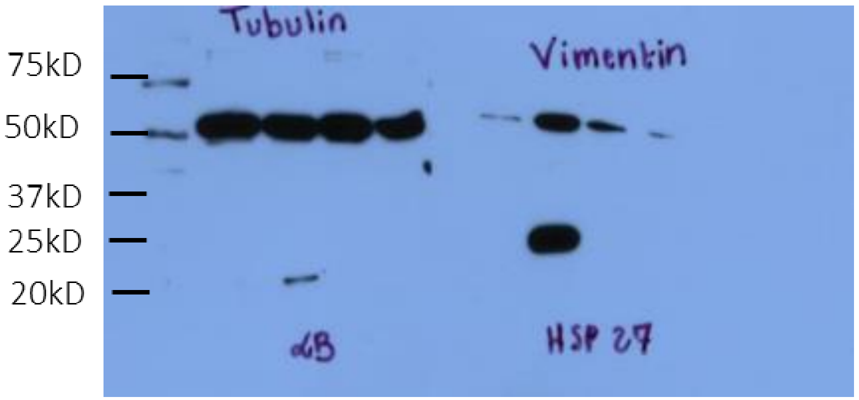

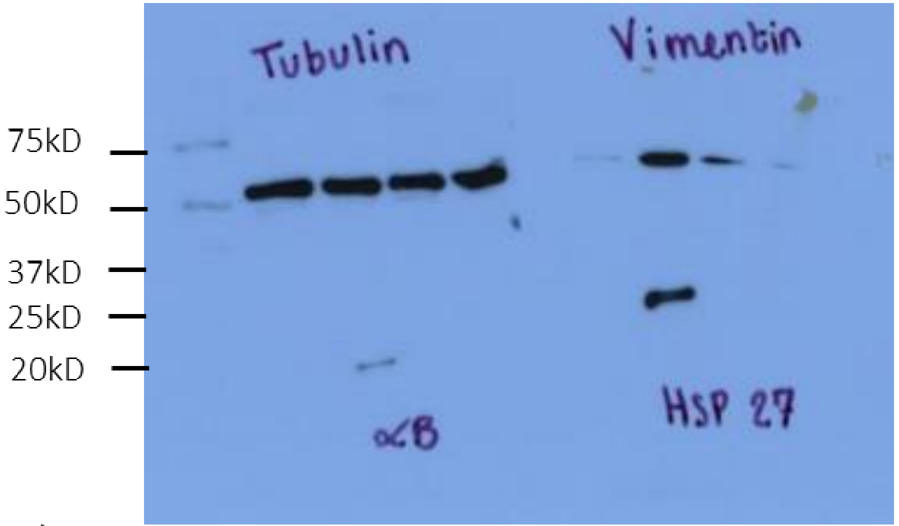

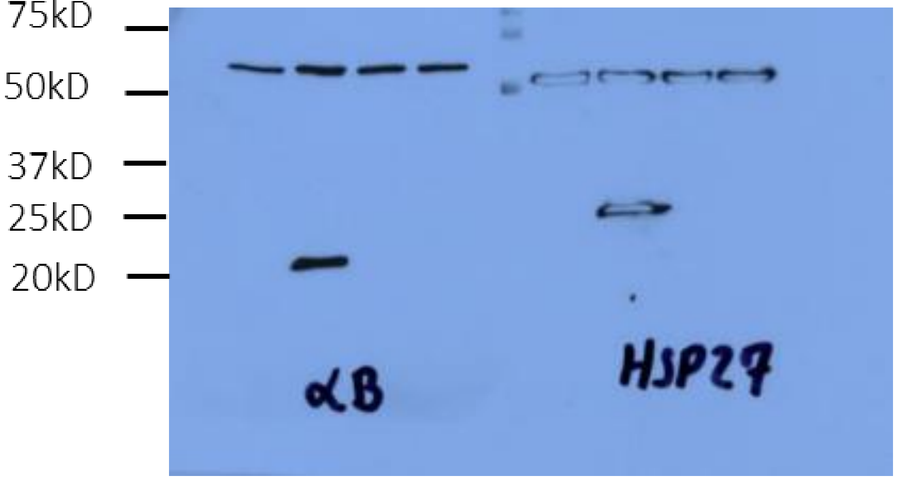

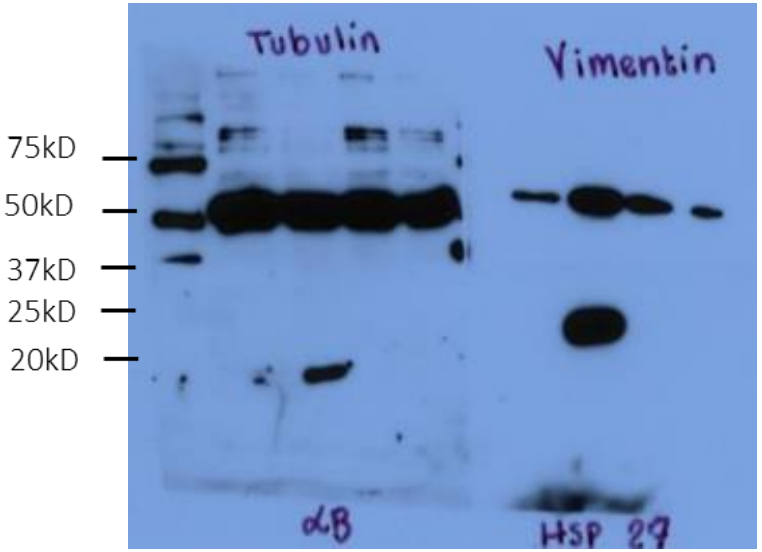

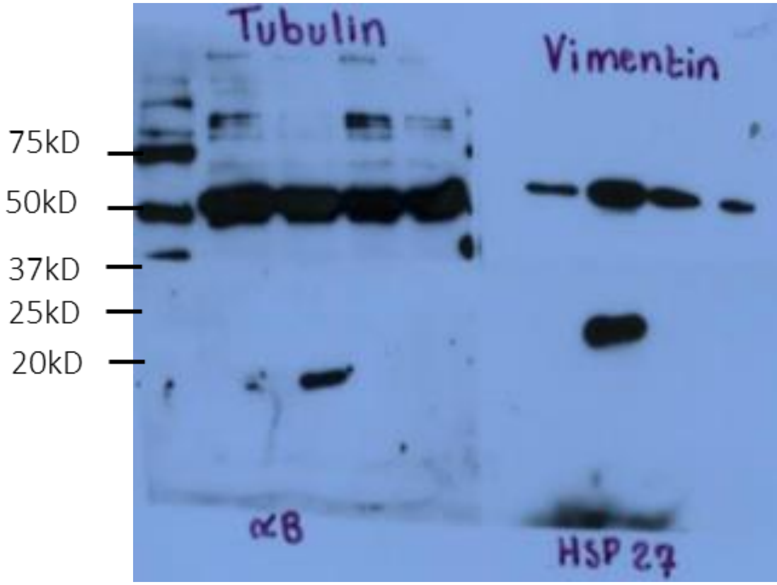

Films_Data OVCAR8R _Fig 1d

Excluded 0.5μM in Figure

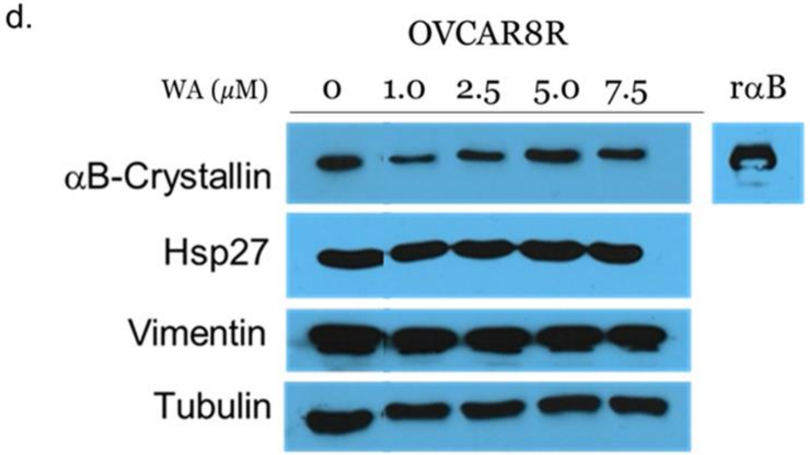

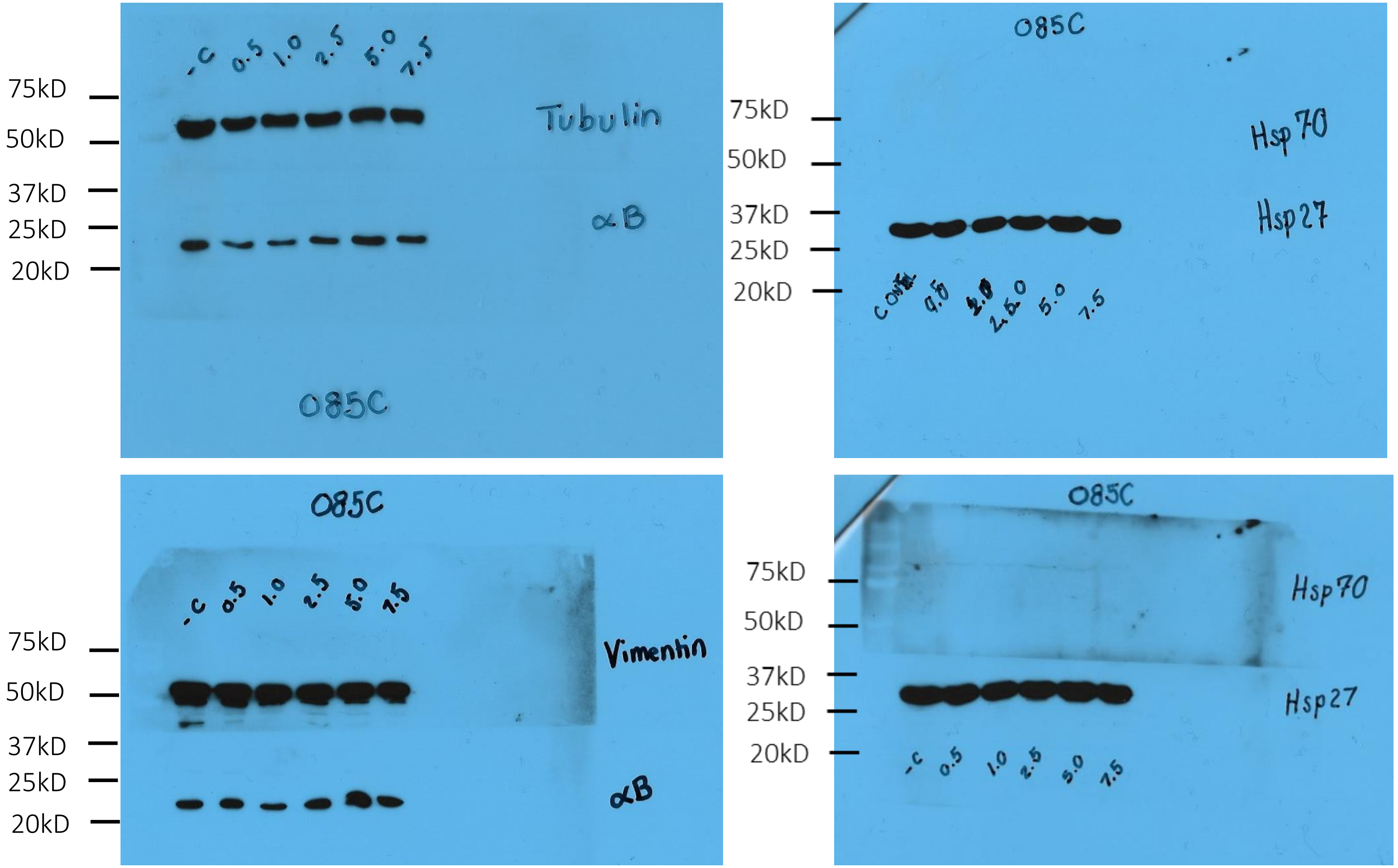

Films_Data OVCAR8 and OVCAR8R _Fig 1c

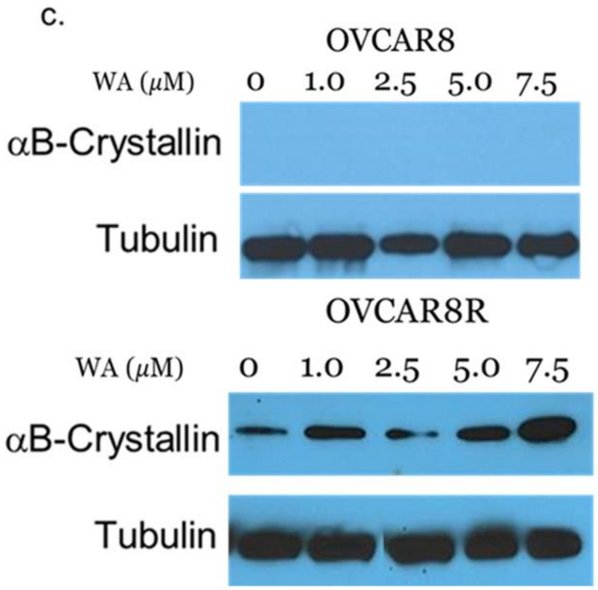

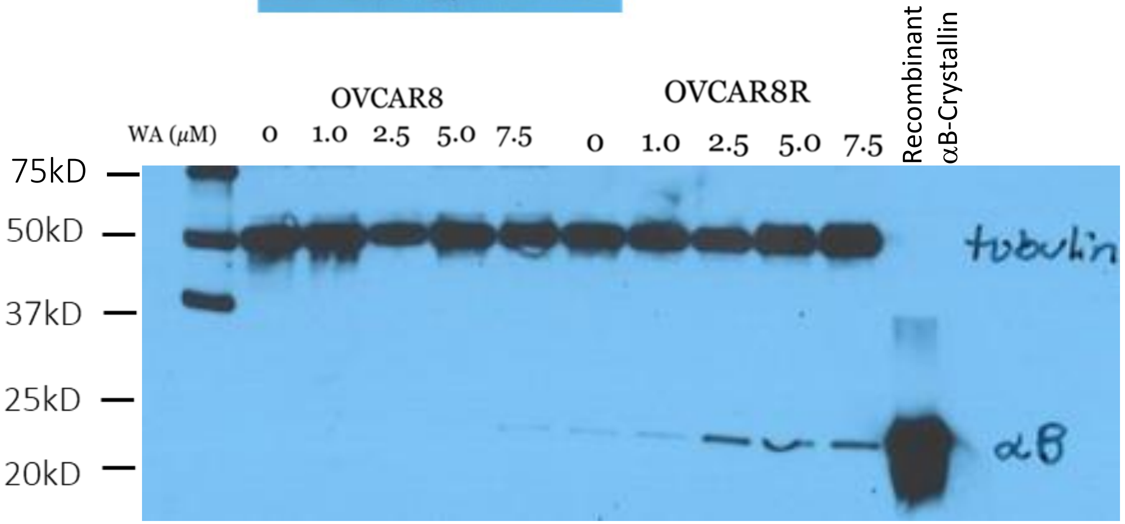

Films_Data OVCAR8 and OVCAR8R _Fig 1c
Rerun of same samples for clarity

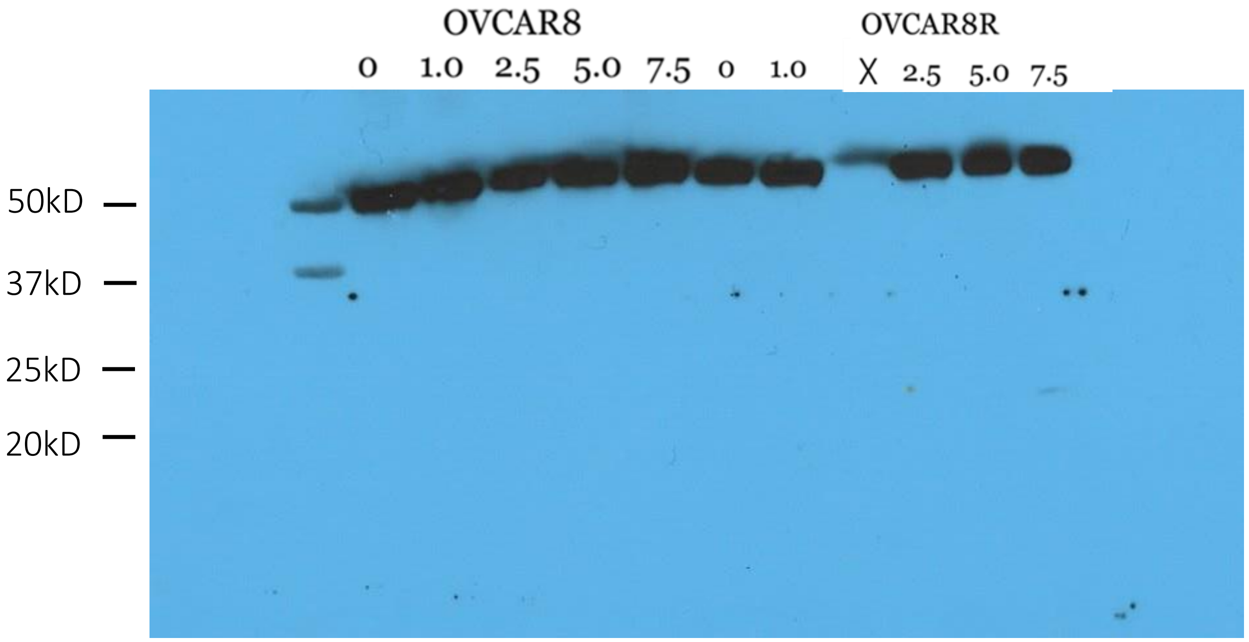

Short exposure, Tubulin 50kD

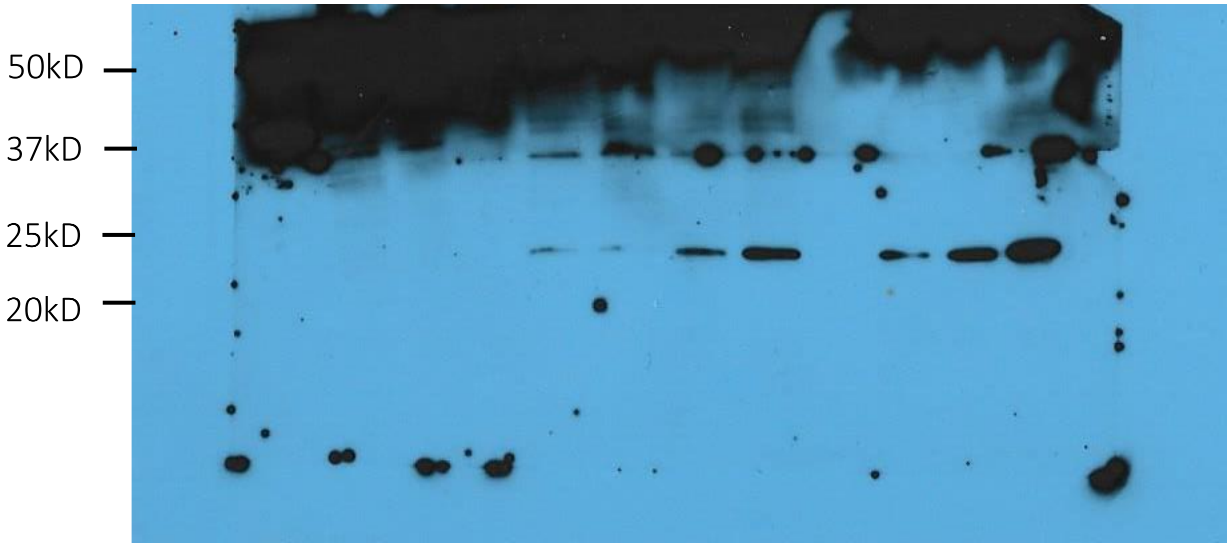

Long exposure, αB-Crystallin 21kD

Films_Data OVCAR8 and OVCAR8R _Fig 1c
X = skipped lane in loading

Films_Data WA Dose_Fig 6 a, b, c, d

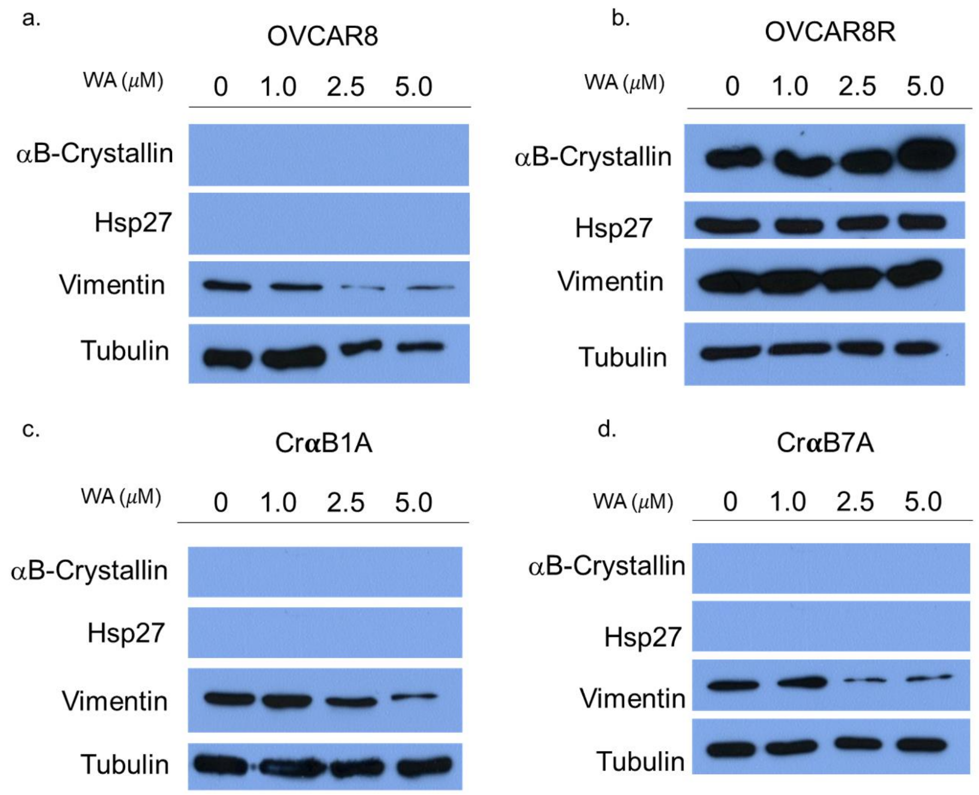

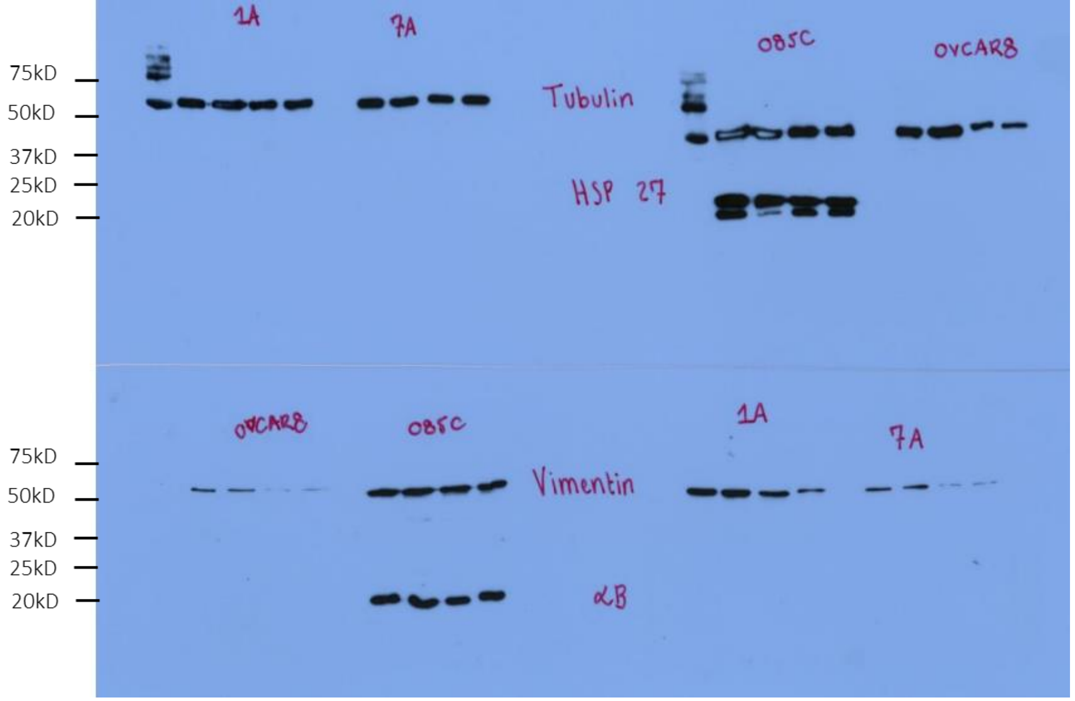

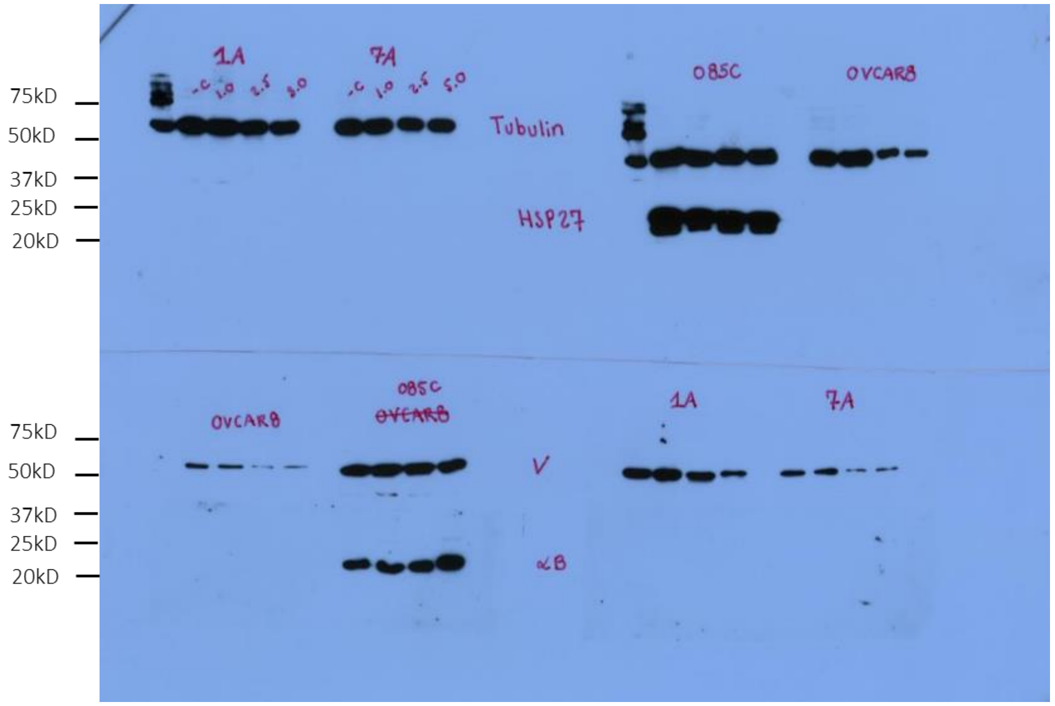

Films_Data_Fig 8c

Excluded OVCAR8R/O85C from last lane with clones 1A_7A

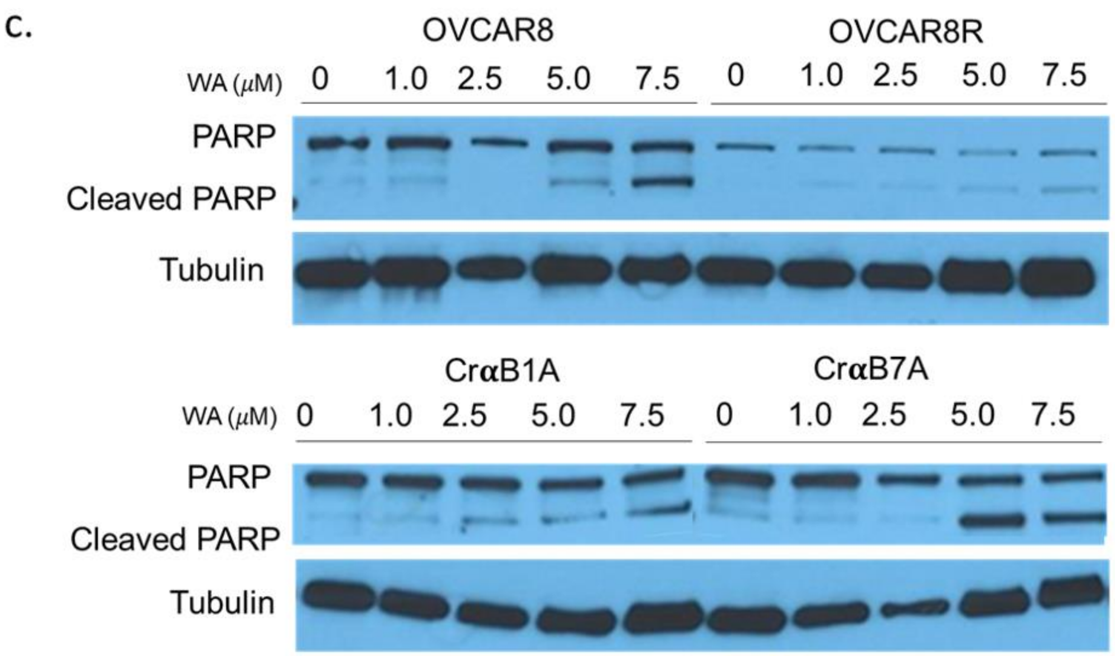

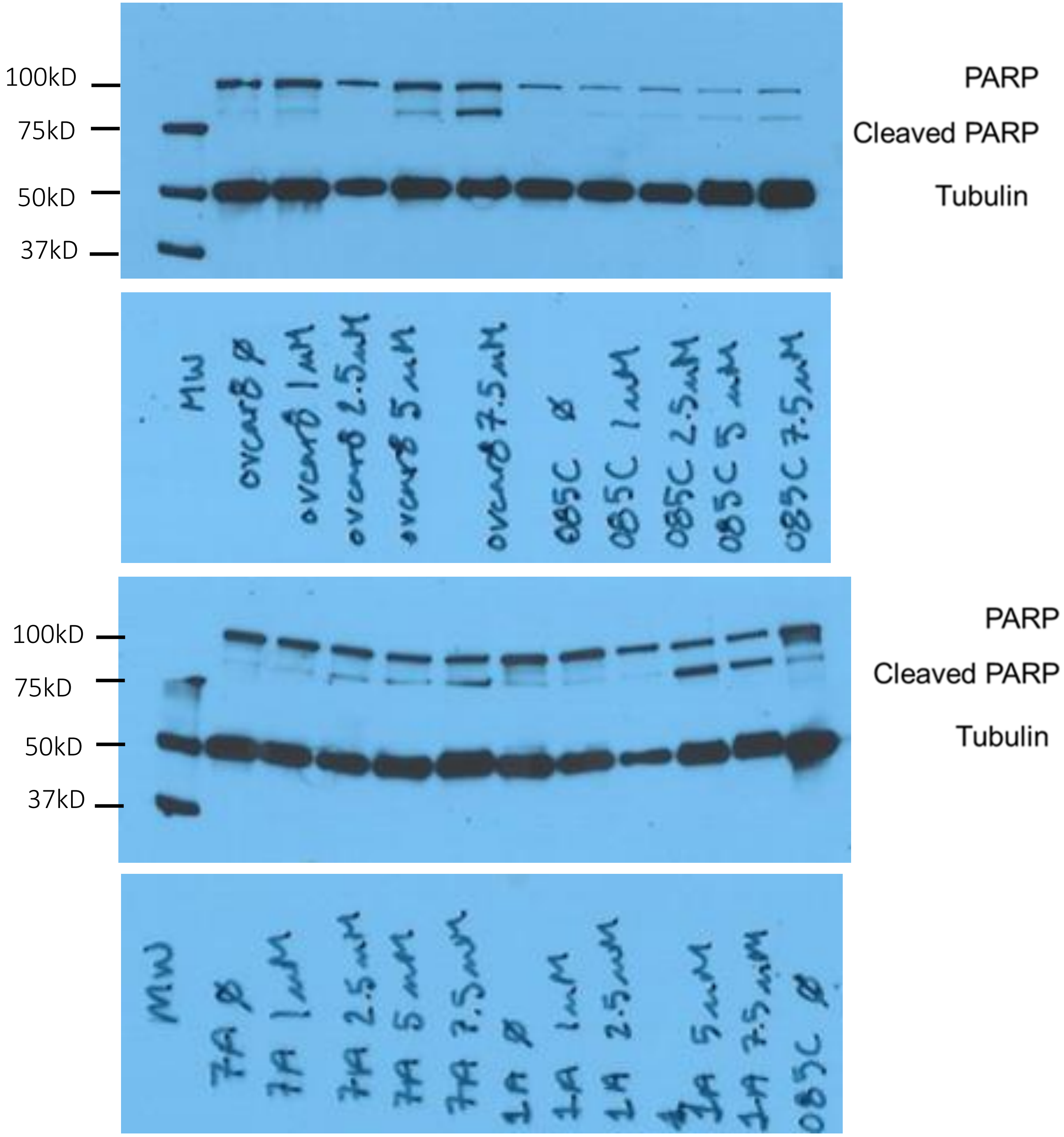

Films_Heat Shock Experiment_S1 Fig

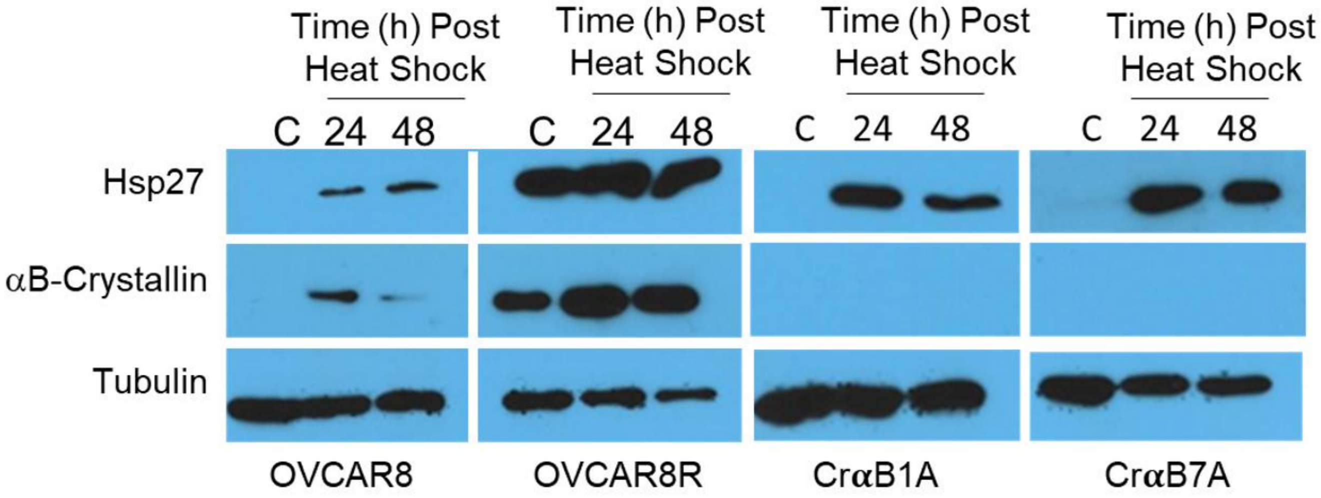

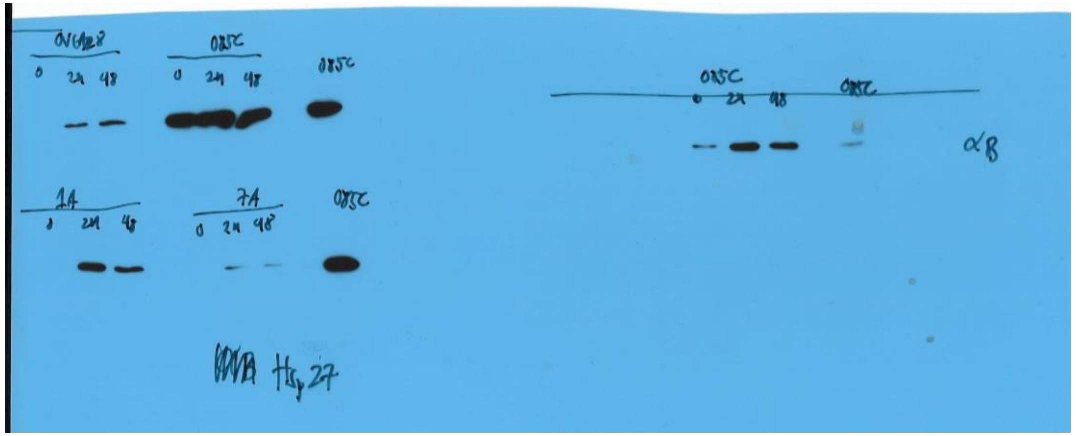

Hsp27

αB-Crystallin

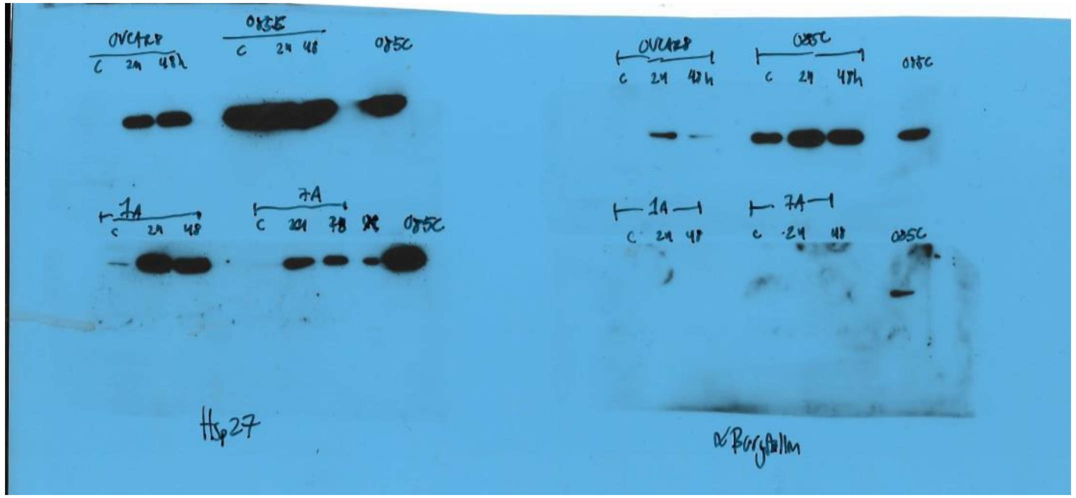

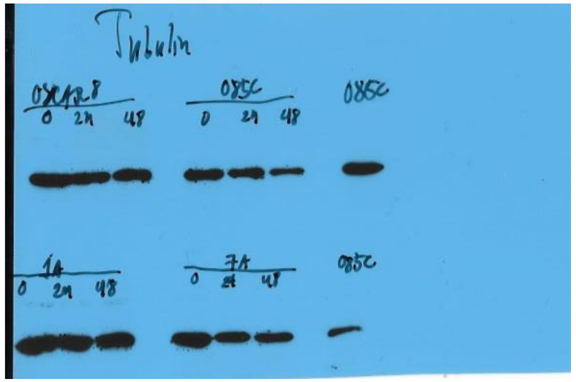

Tubulin
